# Nanostructuring of Mg-Based Hydrogen Storage Materials: Recent Advances for Promoting Key Applications

**DOI:** 10.1007/s40820-023-01041-5

**Published:** 2023-04-10

**Authors:** Li Ren, Yinghui Li, Ning Zhang, Zi Li, Xi Lin, Wen Zhu, Chong Lu, Wenjiang Ding, Jianxin Zou

**Affiliations:** 1 National Engineering Research Center of Light Alloys Net Forming and State Key Laboratory of Metal Matrix Composites, Shanghai Engineering Research Center of Mg Materials and Applications and School of Materials Science and Engineering, Center of Hydrogen Science, Shanghai Jiao Tong University, Shanghai, 200240 People’s Republic of China; 2 Instrumental Analysis Center of SJTU, Shanghai Jiao Tong University, Shanghai, 200240 People’s Republic of China

**Keywords:** Mg-based hydrogen storage materials, Nanostructure, Hydrogen storage, Thermodynamics, Kinetics, On-board hydrogen storage

## Abstract

- A comprehensive discussion of the recent advances in the nanostructure engineering of Mg-based hydrogen storage materials is presented.
- The fundamental theories of hydrogen storage in nanostructured Mg-based hydrogen storage materials and their practical applications are reviewed.
- The challenges and recommendations of current nanostructured hydrogen storage materials are pointed out.

## Abstract (article)

**Abstract:**

With the depletion of fossil fuels and global warming, there is an urgent demand to seek green, low-cost, and high-efficiency energy resources. Hydrogen has been considered as a potential candidate to replace fossil fuels, due to its high gravimetric energy density (142 MJ kg^−1^), high abundance (H_2_O), and environmental-friendliness. However, due to its low volume density, effective and safe hydrogen storage techniques are now becoming the bottleneck for the "hydrogen economy". Under such a circumstance, Mg-based hydrogen storage materials garnered tremendous interests due to their high hydrogen storage capacity (~ 7.6 wt% for MgH_2_), low cost, and excellent reversibility. However, the high thermodynamic stability (ΔH =  − 74.7 kJ mol^−1^ H_2_) and sluggish kinetics result in a relatively high desorption temperature (> 300 °C), which severely restricts widespread applications of MgH_2_. Nano-structuring has been proven to be an effective strategy that can simultaneously enhance the ab/de-sorption thermodynamic and kinetic properties of MgH_2_, possibly meeting the demand for rapid hydrogen desorption, economic viability, and effective thermal management in practical applications. Herein, the fundamental theories, recent advances, and practical applications of the nanostructured Mg-based hydrogen storage materials are discussed. The synthetic strategies are classified into four categories: free-standing nano-sized Mg/MgH_2_ through electrochemical/vapor-transport/ultrasonic methods, nanostructured Mg-based composites via mechanical milling methods, construction of core-shell nano-structured Mg-based composites by chemical reduction approaches, and multi-dimensional nano-sized Mg-based heterostructure by nanoconfinement strategy. Through applying these strategies, near room temperature ab/de-sorption (< 100 °C) with considerable high capacity (> 6 wt%) has been achieved in nano Mg/MgH_2_ systems. Some perspectives on the future research and development of nanostructured hydrogen storage materials are also provided.

**Graphical Abstract:**

## Graphical abstract

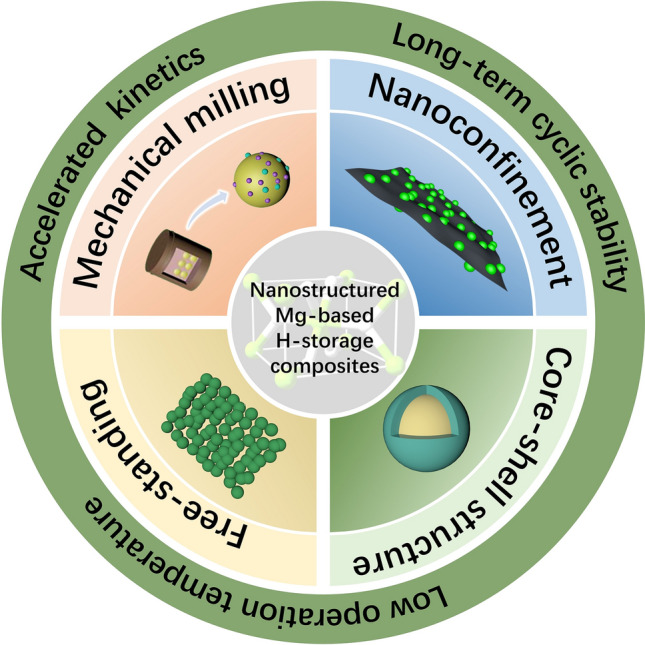

## Introduction

In recent decades, the energy crisis and global warming have promoted a growing demand for renewable clean energy [1, 2, 3]. As a clean and sustainable energy resource, hydrogen (H_2_) has been hailed as a future fuel that holds great promise in replacing ever-being-exhausted fossil fuels and aiding the transition to net-zero emissions [4, 5]. Hydrogen is the most abundant element in the universe and its combustion produces only water [6, 7, 8]. However, many obstacles need to be overcome to realize the so-called “hydrogen economy”. The usage of hydrogen energy includes hydrogen production, hydrogen storage, hydrogen transportation, and hydrogen utilization. As known, the main challenge for the applications of hydrogen energy is the development of suitable approaches for hydrogen storage [9].

Economic, efficient, and safe hydrogen storage methods play a crucial role in exploiting hydrogen energy, reducing carbon emissions, and improving the utilization efficiency of renewable clean energies [10]. Compressed gaseous hydrogen storage technologies have long taken a dominant position in this field due to their simple, convenient, low energy consumption, and efficient advantages [11]. However, high-pressure gaseous hydrogen storage technology has the disadvantage of low volumetric hydrogen storage density and the safety risk of leakage and explosion. Cryogenic liquid hydrogen storage is limited by high cost and energy consumption [12]. Solid-state hydrogen storage in hydrides has been considered as a promising hydrogen storage technology [13]. Although the industrial application of solid-state hydrogen storage technologies with metal hydride is still in the stage of an attack. Metal hydride-based hydrogen storage method can effectively overcome the shortcomings rising from other hydrogen storage techniques, and it is suitable for fuel cell vehicles due to its high volumetric hydrogen storage density, easy operation, convenient transportation, low cost, and high safety [14, 15, 16].

Among numerous hydrides, magnesium hydride (MgH_2_) has received wide attention by virtues of its high gravimetric (7.6 wt% H_2_) and volumetric (110 kg H_2_ m^−3^) hydrogen storage capacity, abundant reserve, and environmental friendliness [17]. However, the implementation of MgH_2_ as a hydrogen-storage medium has long been restricted by two dominating intrinsic challenges, the first obstacle is the high thermodynamic stability (ΔH = 74.7 kJ mol^−1^ H_2_) resulting in the high decomposition temperature of MgH_2_. The desorption enthalpy for MgH_2_ should be tailored into the range of 42–55 kJ mol^−1^ H_2_, if the Mg/MgH_2_ releases hydrogen near 1 bar and fuel-cell operating temperatures (50–150 °C) [18, 19]. Another obstacle is the rather sluggish hydrogen ab/de-sorption kinetics originating from high H_2_ dissociation energy barrier, slow hydrogen diffusion rate in MgH_2_ bulk, etc. [20]. As early as 1951, MgH_2_ was first synthesized by direct reaction of Mg and H_2_. In the past decades, great efforts have been devoted to the development of Mg-based hydrogen storage composites with fast kinetics, low operation temperature, high reversible capacity, long cycling life, low cost, and high safety, as landmarked by the application of alloying [21, 22, 23, 24, 25, 26], nano-structuring [27, 28, 29, 30, 31], and catalyzing [32, 33, 34, 35, 36]. Alloying is a simple and mature method to modify Mg/MgH_2_. By adding alloying elements to the Mg/MgH_2_ system to change its hydrogen ab/de-sorption reaction paths, the thermodynamic properties of MgH_2_ can be effectively improved. In 1968, Reilly et al. [37] first discovered that the intermetallic compound Mg_2_Ni, formed by introducing the alloying element Ni into Mg/MgH_2_ system, presented excellent hydrogen ab/de-sorption thermodynamic performances. Subsequently, Fe, Co, Si, Cu, and other alloying elements were also introduced into Mg/MgH_2_ system to investigate the hydrogen storage properties of their corresponding alloy compounds. However, the biggest drawback of alloying is that the introduction of alloying elements will lead to capacity loss, and some Mg-based hydrogen storage alloys exhibit an irreversible hydrogen ab/de-sorption process. Additionally, it is worth emphasizing that MgH_2_ is a semiconductor with no available electronic states at the Fermi level [38], having almost no catalytic activity toward H_2_ surface reactions. In contrast, The Fermi level of transition metals is located around *s*-type orbitals, which is a necessary condition to promote H_2_ surface reactions [39]. Hence, catalyzing has been a widely studied and efficient method to improve the hydrogen storage kinetics of MgH_2_ since the 1990s. The scope of these catalysts extends from transition metals to transition metal oxides, halides, and carbides. In addition to the catalyzing, the preparation of hydrogen storage composites by compounding complex hydrides with MgH_2_ is also a hot research topic in recent years [40, 41]. Although the alloying and catalyzing could indeed ameliorate the performances of MgH_2_ from the extrinsic perspective, the improvement is restricted by the inevitable agglomeration and growth of the additives during cycling, as well as the low density of exposed active sites. Hence, from an intrinsic point of view, nano-sized MgH_2_ has been widely studied through various nanotechnologies giving rise to improved hydrogen storage performances of MgH_2_ in recent years.

Based on the above discussions, nano-structuring has been regarded as one of the most efficient methods to destabilize MgH_2_ and minimize the decomposition enthalpy among various modification strategies [42, 43]. Nano-sized materials have peculiar properties that could not be expected in the bulk phase. The surface layer atoms of nano-sized materials in the sub-stable state with high surface energy are highly susceptible to combining with other atoms and converting to the stable state, due to the presence of a large number of unsaturated bonds [44, 45]. It is worth emphasizing that the essence of the hydrogen ab/de-sorption process of Mg-based hydrogen storage composites is the bonding and breaking of Mg and H atoms, the nanosizing of MgH_2_ has promising applications in the improvement of thermodynamics and kinetics of Mg-based hydrogen storage materials. Furthermore, it is important to distinguish between grain size, particle size, and crystal size in order to accurately describe the effects of each factor on the kinetics and thermodynamics of MgH_2_. The particle size refers to the size of individual particles of a material. The particle size of MgH_2_ can affect its thermodynamics and kinetics by altering the surface area-to-volume ratio. In general, smaller particles have a larger surface area-to-volume ratio than that of larger particles, which can enhance the reactivity and reaction rate of MgH_2_ by increasing the number of active sites available for de/re-hydrogenation. The crystal size of MgH_2_ refers to the size of individual crystals in a material. When MgH_2_ is in a crystalline state, the size of the crystals can impact the nucleation and growth of new phases, as well as the overall crystal structure of the material, which can affect the reaction kinetics and thermodynamics. In general, smaller crystal sizes can promote faster nucleation and growth of new phases, as well as increase the number of defects and grain boundaries that can serve as reaction sites. Furthermore, the downsizing of the MgH_2_/Mg crystal leads to a change in the lattice, which will result in a change in desorption energy via the changed lattice energies, thereby destabilize MgH_2_. The grain size of MgH_2_ is related to the crystal size, but refers specifically to the size of the individual grains in a polycrystalline material. In the case of MgH_2_, smaller grains can provide a greater surface area, more nucleation sites, and shorter diffusion path for hydrogen absorption and desorption, similar to smaller crystal sizes. Thus, the main reasons for the amelioration of the hydrogen storage properties of Mg-based materials by nanosizing are as follows:

- With the increase of the specific surface area of nano-sized MgH_2_, the contact area between the matrix and hydrogen is increased, accelerating the diffusion rate of hydrogen to the matrix. Besides, the contact area between the MgH_2_/Mg and catalysts is also increased, improving catalytic efficiency.
- With the nanosizing of MgH_2_, the surface energy increases, facilitating the adsorption of H_2_ on the surface of Mg particles and the destabilization of MgH_2_.
- The high density of grain boundaries among nanoparticles provides more diffusion paths for hydrogen atoms, improving the hydrogen storage kinetics of MgH_2_.
- Reducing the size of MgH_2_ effectively shortens the diffusion path of hydrogen, enhancing the kinetic properties of MgH_2_/Mg.

The preparation methods of nano-sized Mg-based hydrogen storage materials include ball-milling, vapor deposition method, plasma metal reaction, chemical reduction of Mg precursors, and nanoconfinement. Many high-quality reviews have been published in the last decade covering the thermal destabilization and catalytic tuning of the kinetics of Mg-based hydrogen storage materials [14, 20, 46, 47]. However, a specific focus on nanostructure engineering of Mg-based hydrogen storage materials is still missing and necessary for their applications in future energy storage.

**Scheme 1:**
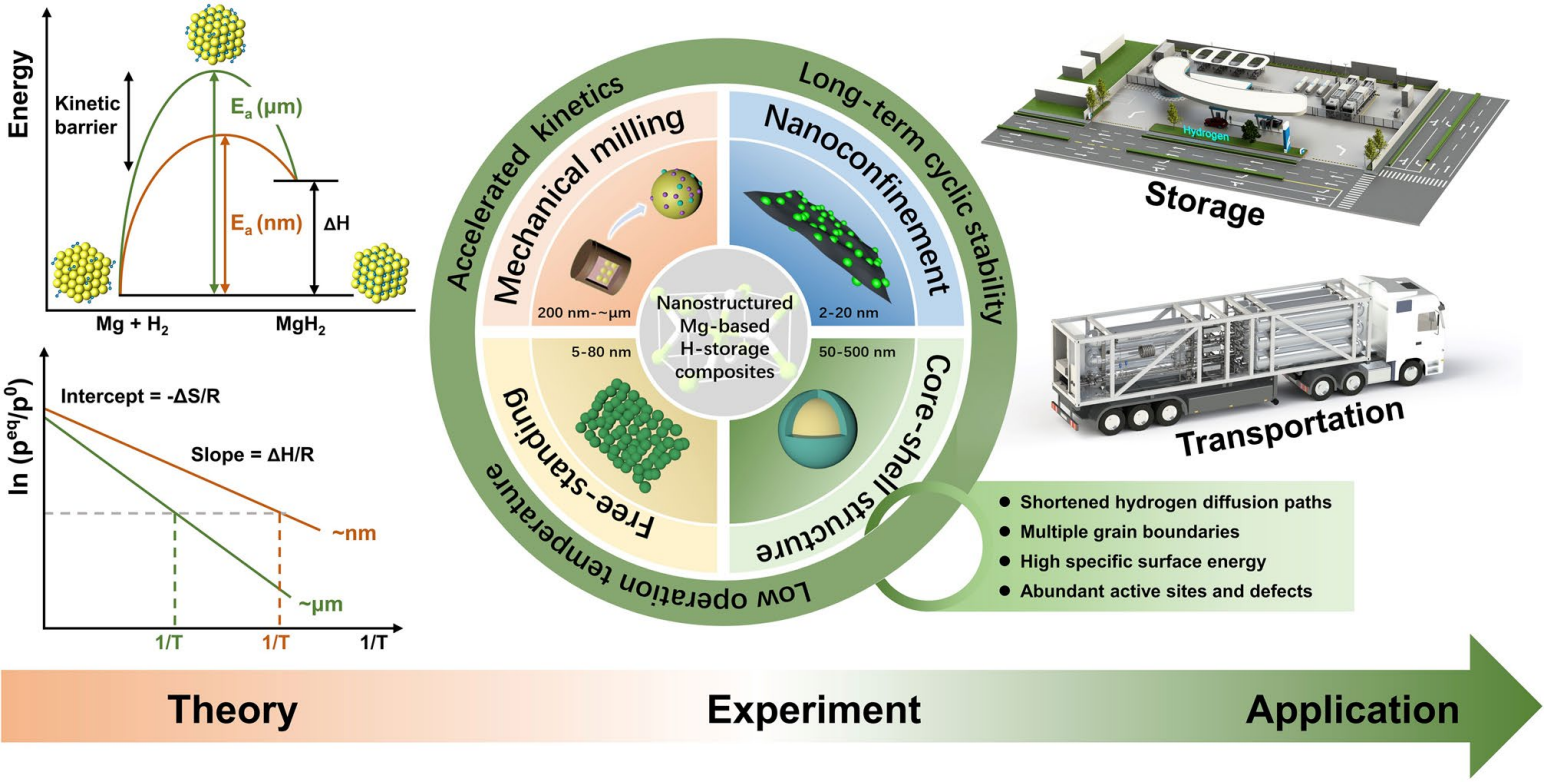
Schematic illustrations of fundamental theories and synthetic strategies for nano-engineering of Mg-based hydrogen storage materials

Herein, we make great efforts to present a comprehensive overview of fundamental theory and synthetic methodologies for intricate nanostructured Mg-based hydrogen storage composites, as depicted in Scheme 1. The fundamental theories of hydrogen storage regarding MgH_2_ are highlighted with special emphasis on thermodynamics, kinetics, and cycling stability. This review paper summarizes the latest trends in the design of nanostructured Mg-based hydrogen storage materials, important breakthroughs in the field, and the challenges for Mg-based composites applied in the commercial energy conversion and storage devices.

## Fundamental Theories of Hydrogen Storage in MgH_2_

### Thermodynamics and Destabilization of the Mg/MgH_2_ System

**Fig. 1:**
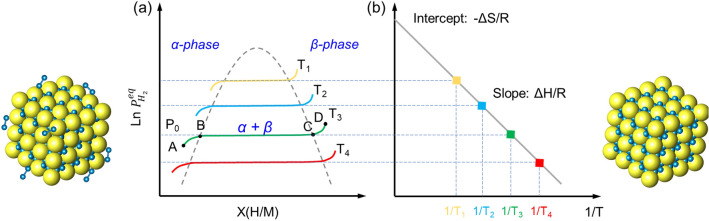
**a** Pressure–composition isotherm plot of ${\text{Mg }} + {\text{H}}_{{2}} \rightleftarrows {\text{MgH}}_{{2}}$ transition. **b** van't Hoff plot related to the phase transition of ${\text{Mg }} + {\text{H}}_{{2}} \rightleftarrows {\text{MgH}}_{{2}}$. The enthalpy and entropy of hydrogenation and dehydrogenation could be obtained from the slope and intercept, respectively. Schematic representations of the α-phase (left) and β-phase (right) of metal hydride are also presented

The hydrogen ab/de-sorption process of hydrides is a dynamic equilibrium of three phases: hydrogen, metal, and the corresponding hydride (Fig. 1). As shown in Fig. 1a, hydrogen pressure, composition, and temperature are the crucial factors determining the phase equilibrium [48]. During the process of isothermal hydrogenation, a solid solution (α-phase) is first formed. With the increase of hydrogen pressure, the solid solution starts to transform into hydride (β-phase), the β-phase nucleates and grows, and the hydrogen pressure remains unchanged as the phase transformation proceeds. Until the α-phase completely transforms to the β-phase, the hydrogenation reaction completes. By calibrating the pressure–composition–temperature equilibrium point in the process of hydrogen ab/de-sorption, the PCT curve can be obtained (Fig. 1b). The relationship between plateau pressure (*P*_*eq*_) and temperature (T) in the PCT curve can be described by the van't Hoff equation:

2-1 $$\ln \left( {\frac{{p_{eq} }}{{P_{0} }}} \right) = \frac{\Delta H}{{RT}} - \frac{\Delta S}{R}$$

In this formula, *P*_0_ is the atmospheric pressure (1.01 × 10^5^ Pa); *∆H* and *∆S* are the enthalpy and entropy of the hydrogen ab/de-sorption, respectively; and *T* is the absolute temperature; *R* is the gas constant (*R* = 8.314 J mol^−1^ K^−1^). According to the linear fitting between ln*P* and 1000/*T*, *∆H* and *∆S* can be calculated. Notably, the value of the re/de-hydrogenation enthalpy (*∆H*) is an important indicator to measure the strength of the Mg–H bond. The larger the absolute value of *∆H* is, the stronger the Mg–H bond will be.

The thermodynamic stability of Mg/MgH_2_ system is mainly determined by the feature of the Mg–H bond. The Mg–H bond in the Mg/MgH_2_ system is covalent–ionic mixed [49], having a relatively high bonding energy of around 3.35 eV. The nature of Mg–H bond results in a high enthalpy for the decomposition of MgH_2_, which is around 74.7 kJ for releasing 1 mol of H_2_ [50], leading to a high temperature for hydrogen desorption. Under atmospheric pressure, MgH_2_ starts to release H_2_ at a temperature of 280 °C, which is far from the requirements of practical applications [51]. Many efforts have been made to thermodynamically destabilize the Mg/MgH_2_ systems, such as alloying of Mg with other elements, inducing the formation of metastable γ-MgH_2_ phase, and nano-structuring [52]. Cheung et al. [53] simulated the relationship between grain size reduction and decreased structural stability. They concluded that significant changes in the thermodynamics of hydrogen desorption are observed only if the grain size is reduced to below 2 nm.

### Kinetics of the Mg/MgH_2_ System

The thermodynamic parameters of the hydrogen storage materials characterize the driving force of phase transformation during hydrogen absorption and desorption. While hydrogen storage performances of the hydrides are also related to the re/de-hydrogenation rate, i.e., the kinetic properties.

**Fig. 2:**
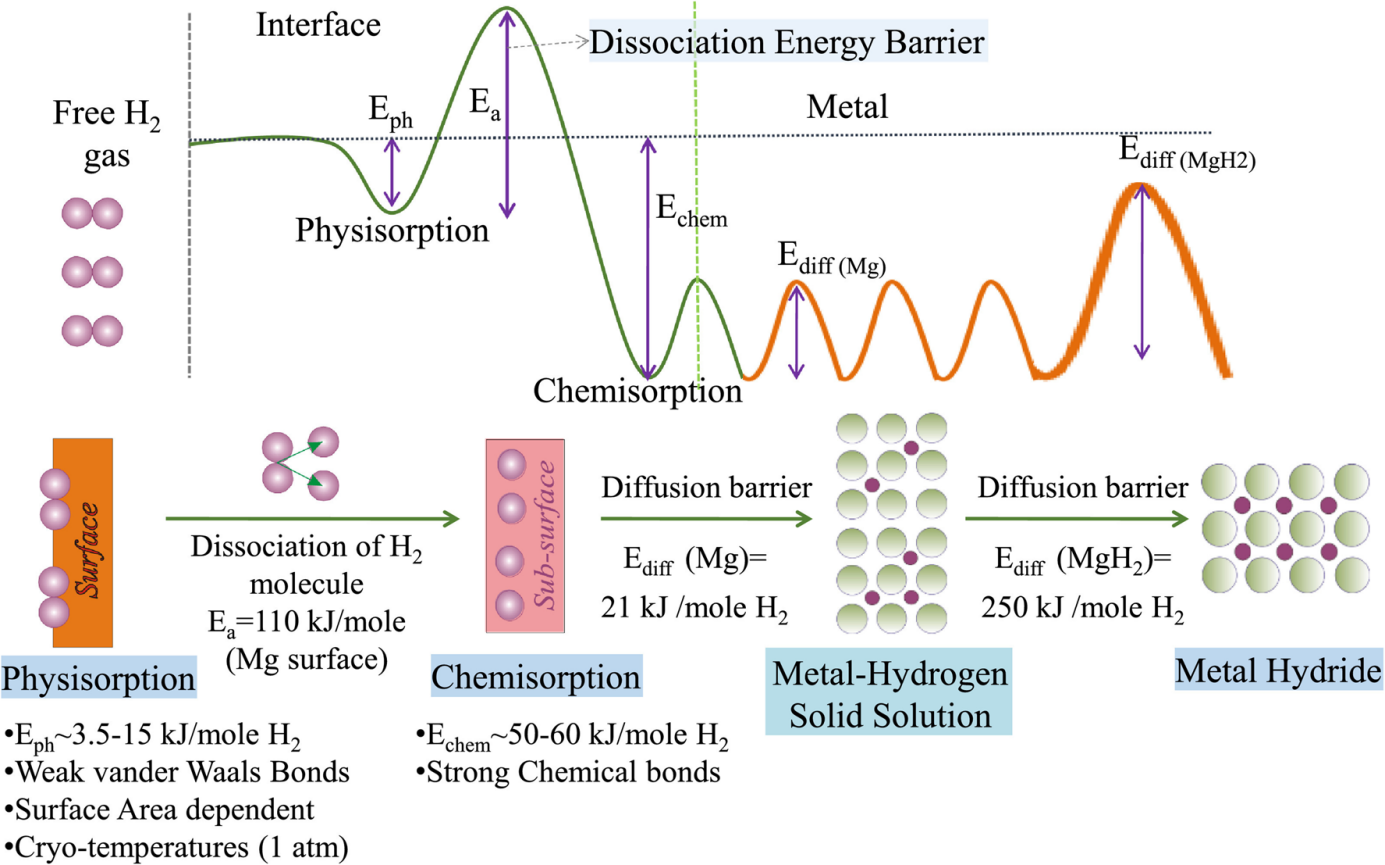
Schematic diagram describing continuous energy barriers in the process of hydrogen absorption (up) and illustration of the kinetic steps in the hydrogen storage process (down). Reproduced with permission from Ref. [54]. Copyright 2021 Elsevier

The hydrogenation process of Mg includes four main steps: H_2_ physisorption, H_2_ dissociation, H chemisorption, and H atom diffusion [55]. Each step needs to overcome an energy barrier, called the reaction activation energy (Fig. 2). The relevant experiments have revealed that the rate-determining step is the nucleation-growth of MgH_2_, which is determined by the hydrogen diffusion rate in Mg lattice [56]. However, the diffusivity of hydrogen in Mg lattice is quite low, which is measured to be 10^−20^ m^2^ s in bulk Mg and 10^−18^ m^2^ s along grain boundaries [57, 58]. It is worth emphasizing that the firstly formed MgH_2_ will act as a barrier to further diffusion of hydrogen into bulk Mg, limiting the reaction rate of the hydrogenation process [59]. The hydrogen desorption of MgH_2_ is mainly determined by the breakage of Mg–H bonds, diffusion of hydrogen atoms in the bulk phase, and recombination of hydrogen atoms. Accordingly, it is demonstrated that the sluggish kinetics (160 kJ mol^−1^ H_2_) severely limits the wide application of Mg/MgH_2_ system. Particularly, in the dehydriding stage of coarse particles, successively formed fresh Mg at the surface layer may function as the diffusion barrier to the hydrogen escaping from MgH_2_. However, in nano-sized MgH_2_ particles, the Mg phase may form simultaneously all through the material and the entire process of desorption is then governed by fast hydrogen diffusion rather than slow Mg–MgH_2_ boundary movement. In other words, particle size of MgH_2_ has a profound effect on its hydrogen storage kinetic properties [60, 61].

To understand the mechanism of reaction kinetics, it is necessary to analyze kinetic models in the process of hydrogen ab/de-sorption. The kinetic models of hydrogen storage materials in the process of de/re-hydrogenation can be summarized in Table 1 [62, 63, 64].

**Table 1:** Kinetic models for hydrogen absorption and desorption process

Model equation	Description
$$\alpha = kt$$	
$[ - \ln (1 - \alpha )]^{1/n} = kt$	JMAK model n = 3, three-dimensional nucleation and growth mode n = 2, two-dimensional nucleation and growth mode
$1 - \ln (1 - \alpha )^{1/n} = kt$	Shrinking core model n = 3, three-dimensional nucleation and growth mode n = 2, two-dimensional nucleation and growth mode
$1 - \left( {\frac{2\alpha }{3}} \right) - (1 - \alpha )^{\frac{1}{3}} = kt$	Shrinking core model Three-dimensional growth process of the nucleus is controlled by the diffusion process Slowed growth rate of the grain boundary interface affects the growth rate of the nucleus

Among them, the hydrogen ab/de-sorption process of most hydrogen storage materials can be described by the nucleation and growth mechanism (JMAK model), and the equation is as follows [65]:

2-2 $$\ln \left[ { - \ln \left( {1 - \alpha } \right)} \right] = n{\text{ln}}k + n{\text{ln}}t$$

In this formula, *k* is the rate constant of hydrogen ab/de-sorption; *α* is the reaction fraction corresponding to *t*; *n* is the dimension determining the abstract model. By establishing the linear relationship between $\ln \left[ { - \ln \left( {1 - \alpha } \right)} \right]$ and ${\text{ln}}t$, the value of *n* can be obtained from the slope, and the reaction rate constant of *k* can be calculated by the intercept.

Furthermore, the activation energy (*E*_*a*_) of hydrogen storage materials in the process of hydrogen ab/de-sorption can be obtained through the Arrhenius formula [66]:

2-3 $$k = Ae^{{ - \frac{{E_{a} }}{Rt}}}$$

2-4 $${\text{ln}}k = - \frac{{E_{a} }}{RT} + {\text{ln}}A$$

In which *k* is the rate constant, *A* is the pre-exponential factor, and *E*_*a*_ is the activation energy. Thus, the activation energy can be obtained by fitting ln*k* verses 1/*T* [the reaction rate constants of *k* at different temperatures can be calculated from Eq. (2-2)]. The intrinsic mechanism of the hydrogen ab/de-sorption kinetic properties of materials can then be explained from the perspective of activation energy.

Moreover, Kissinger’s method is also usually used to calculate the dehydrogenation activation energy of hydride, typically using differential scanning calorimetry (DSC) experiments, following Kissinger’s equation [66, 67]:

2-5 $$\ln \left( {\frac{\beta }{{T_{p}^{2} }}} \right) = - \frac{{E_{a} }}{{RT_{p} }} + C$$

In this formula, *β* is the heating rate; *T*_*p*_ is the peak temperature; *R* is the gas constant (*R* = 8.314 J mol^−1^ K^−1^); *C* is a constant. By fitting the linear relationship between $\ln \left( {\frac{\beta }{{T_{p}^{2} }}} \right)$ and 1000/*T*_*p*_, the activation energy (*E*_*a*_) can be obtained using DSC results at different heating rates. Kissinger’s method has advantages of being simple and requiring few tests. However, only a single rate-limiting step is assumed, the application of the equation is limited.

**Fig. 3:**
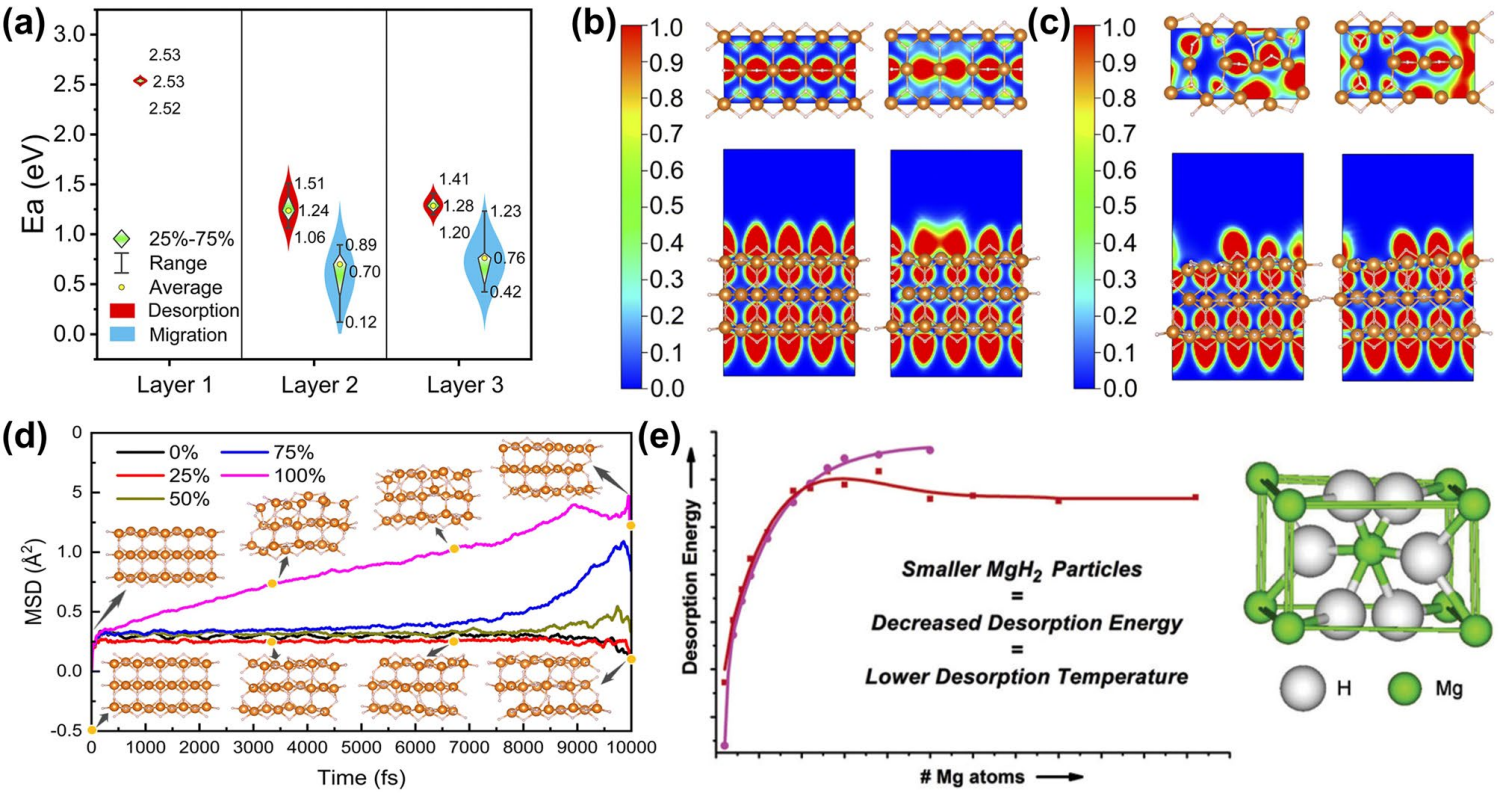
**a** Comparison of the three-layer atomic H migration and dehydrogenation energy barriers. The calculated ELFs of MgH_2_ before the dehydrogenation **b** in processes 1 and 2 (the first layer) and **c** in processes 3 and 4 (the second layer). **d** AIMD simulations on MgH_2_ (110) before and after surface H loss. Yellow dots are the selected points of MgH_2_ before (0%) and after (100%) surface H loss at 500 K. White and orange spheres represent H and Mg, respectively. Reproduced with permission from Ref. [19]. Copyright 2022 Royal Society of Chemistry. **e** Calculated desorption energies for MgH_2_ clusters with both the HF method and DFT method. Reproduced with permission from Ref. [68]. Copyright 2005 American Chemical Society. (Colour figure online)

Dong et al. [19] studied the sequential MgH_2_ dehydrogenation mechanism by analyzing the kinetic and structural changes during the layer-by-layer hydrogen desorption process through spin-polarized density functional theory calculations with van der Waals corrections (DFT-D3). The results showed that initial dehydrogenation barriers (2.52 and 2.53 eV) were much higher than the subsequent reaction barriers (0.12–1.51 eV) (Fig. 3a). Moreover, after the desorption of all surface atomic H, the degree of electron localization in this region dropped sharply, resulting in a burst effect (Fig. 3b, c). The results of AIMD simulations indicated that after the loss of all the surface atomic H, the atomic H of MgH_2_ was inclined to diffuse, and therefore, the dehydrogenation kinetics could be significantly improved (Fig. 3d), which inspired us the importance of promoting the initial dehydrogenation by structural engineering (such as nanostructure engineering) to facilitating the hydrogen desorption of MgH_2_. It was also demonstrated that the desorption energy of MgH_2_ decreased as the cluster size was reduced to below 19 Mg atoms [68]. For the smallest possible cluster, the desorption energy of MgH_2_ dropped even to negative values, which means that the nanostructure engineering makes MgH_2_ unstable (Fig. 3e).

Compared with the improvement in thermodynamics, the kinetic properties of Mg/MgH_2_ can be adjusted more easily and effectively. Numerous strategies are effective in improving kinetic performance, such as alloying, catalyzing, nano-structuring, etc., which will be discussed in the following sections.

### Cycling Stability of the Mg/MgH_2_ System

Long-term cycling stability also plays a decisive role in the application of Mg-based hydrogen storage composites. The experimental results show that the long-term re/de-hydrogenation cycle at high temperatures will deteriorate the hydrogen storage capacity and the hydrogen ab/de-sorption rate [43, 69, 70, 71]. The reasons for the degradation of the system can be attributed to the following two factors. On one hand, the passivation of the Mg/MgH_2_ interface resulted from the reaction between Mg/MgH_2_ and contamination gas in H_2_, causing the loss of capacity [72]. On the other hand, the agglomeration and growth of nanoparticles driven by the interfacial energies increase the diffusion pathway for hydrogen and deteriorate the kinetics of Mg/MgH_2_ systems.

To improve the cyclic stability, apart from using purified H_2_ to prevent capacity loss, the nanoconfinement and encapsulation strategy are effective methods to restrict the agglomeration and growth of nanoparticles. The research of nanoconfinement will be discussed in detail in the following section.

## Tuning the Properties of the Mg/MgH_2_ System Through Nanotechnology

The destabilization of MgH_2_ by nano-structuring has been widely investigated both theoretically and experimentally. As the size of particles reduces to nanoscale, the surface energy cannot be ignored. Due to the extra interfacial free energy stored at the boundary, the hydrogen ab/de-sorption temperature will be decreased, indicating that Mg-based hydrogen storage materials can be substantially destabilized by inducing nanocrystalline structure [73]. The equilibrium pressure of nano-sized MgH_2_ particles and corresponding bulk MgH_2_ during the hydrogen desorption process obeys the following relationship [51]:

3-1 $${\text{ln}}\frac{{P_{nano}^{eq} }}{{P_{bulk}^{eq} }} = \frac{1}{RT}\left( {\frac{{3V_{{{\text{Mg}}}} \gamma_{{{\text{Mg}}}} }}{{r_{{{\text{Mg}}}} }} - \frac{{3V_{{{\text{MgH}}_{2} }} \gamma_{{{\text{MgH}}_{2} }} }}{{r_{{{\text{MgH}}_{2} }} }}} \right)$$

In this formula, $P_{nano}^{eq}$ and $P_{bulk}^{eq}$ are equilibrium pressures of nanoparticles and corresponding bulk, respectively; *V* is molar volume; *γ* is the surface energy density; *r* is radius of the spherical particle. It can be deduced that part of the formation enthalpy will be stored as excessive surface energy when the contribution of the size effect becomes sufficiently large, resulting in the destabilization of the MgH_2_ nanoparticles. Particularly, as a typical phase transformation, the hydrogen ab/de-sorption process first takes place along the interface region, thus interfacial energy plays a crucial role in tuning the hydrogen storage performance. The formation of nanocrystalline with high density of interfaces will induce high extra energy stored in the interfacial region, which can reduce the energy barrier of de/re-hydrogenation [73, 74].

Thus, nano-structuring can significantly reduce the hydrogen ab/de-sorption temperature and increase the rate of re/de-hydrogenation of MgH_2_, due to the introduction of defects, shortening of hydrogen diffusion paths, increasing of nucleation sites, and destabilization of Mg–H bonding. However, due to the high surface energy, nanoparticles are susceptible to agglomeration and growth. It is essential for exploring an appropriate strategy to prepare Mg-based nanostructured composites. In the following sections, we will review the synthesis methods of nanostructured Mg-based hydrogen storage materials in detail.

### Synthesis of Free-Standing Nano-sized Mg/MgH_2_

Wagemans et al. [68] theoretically investigated the influence of crystal grain size on the thermodynamic stability of Mg/MgH_2_. The results showed that both MgH_2_ and Mg become less stable with the decreasing of cluster size and the absolute value of enthalpy reduced dramatically when crystallite sizes were decreased down to less than 1.3 nm. Particularly, a lower decomposition enthalpy of 63 kJ mol^−1^ H_2_, corresponding to a desorption temperature of only 200 °C at 1 bar hydrogen pressure, may be obtained for 0.9 nm sized MgH_2_ crystallites. They indicated that the downsizing of the MgH_2_/Mg caused a change in the lattice, thus resulting in the reduction of desorption energy via the changed lattice energies. Impressively, they found that nano-sized Mg possessed the potential to uptake a few additional percent of hydrogen above the stoichiometric MgH_2_ through a stepwise calculation on the hydrogen sorption processes. The extra hydrogen (10-15%) was not dissociated and adsorbed to the surface of MgH_2_ as hydrogen molecules, depending on the specific surface area of nano-sized MgH_2_, which was rare in bulk systems. It is worth emphasizing that “excess” hydrogen is less strongly bound to the MgH_2_ structure and can therefore be desorbed at lower temperatures. Their quantum chemical study inspires us that it is possible to prepare MgH_2_ clusters that can adsorb extra hydrogen molecules on the surface due to its unique nano-structure, which will release hydrogen at lower temperatures, and is suitable for the operation of proton exchange membrane fuel cell (PEMFC). While the waste heat released by the PEMFC can be used for the dehydrogenation of stoichiometric MgH_2_, thereby achieving maximum energy utilization. However, these assumptions are simply not possible in bulk materials.

**Fig. 4:**
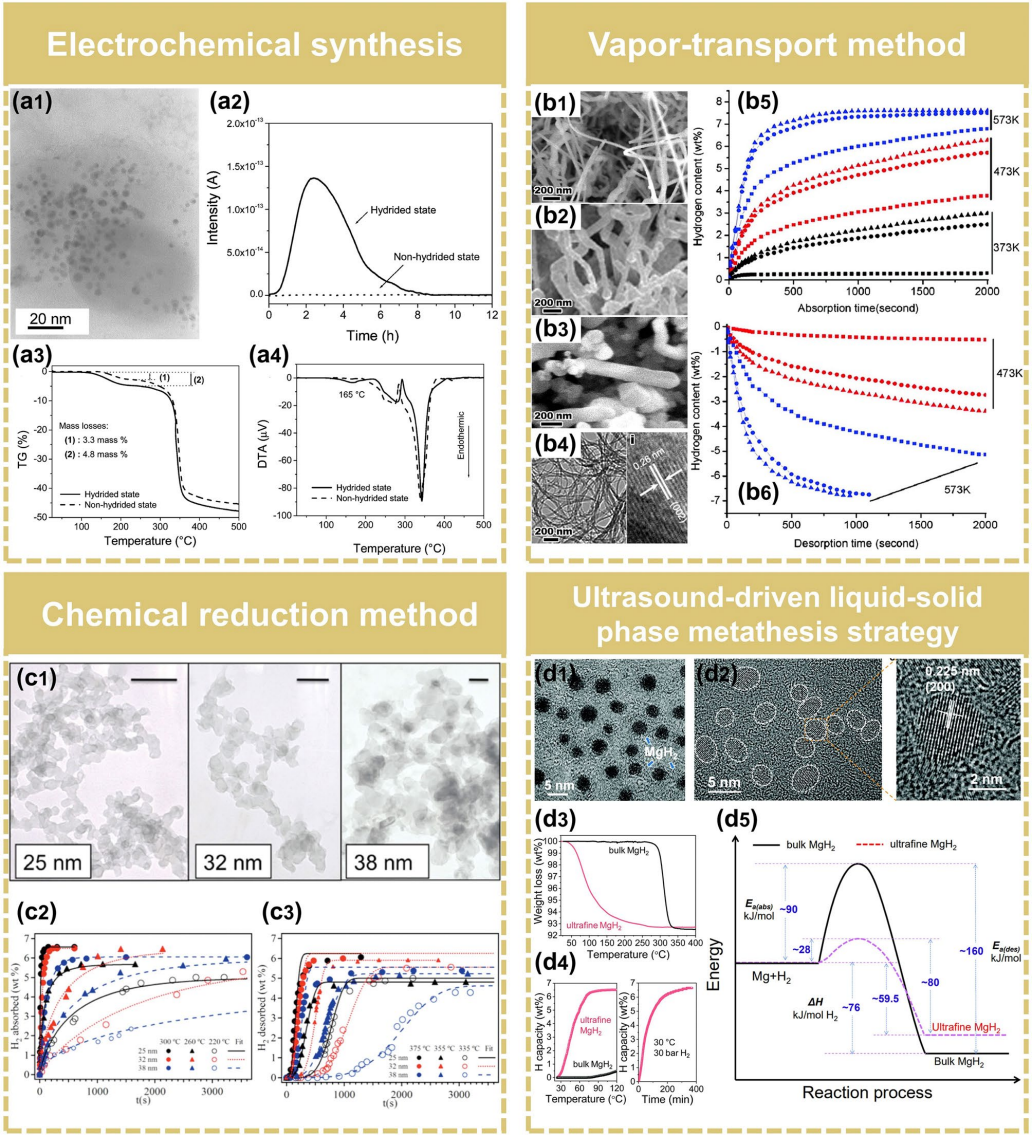
Synthesis of the free-standing nano-sized Mg/MgH_2_. **a1** TEM image of the Mg colloid synthesized by electrochemical method. **a2** Mass spectrometry of H_2_ desorption for the MgH_2_ (hydrided state) and the Mg colloids (non-hydrided state) at 85 °C. **a3**, **a4** TG-DSC signals of the Mg colloid after H_2_ absorption (hydrided sate) and desorption (non-hydrided state). Reproduced with permission from Ref. [75]. Copyright 2008 American Chemical Society; **b1**–**b3** SEM images of Mg nanowires with a diameter of 30–50 nm, 80–100 nm and 150–170 nm, respectively. **b4** TEM and HRTEM images of Mg nanowires with a diameter of 30–50 nm. **b5** Hydrogen absorption and **b6** desorption curves of the Mg nanowires with different diameters (30–50 nm, triangle; 80–100 nm, circle; 150–170 nm, square). Reproduced with permission from Ref. [77]. Copyright 2007 American Chemical Society; **c1** TEM images of Mg nanocrystal samples (scale bar = 100 nm). **c2** Hydrogen absorption and **c3** desorption curves of the Mg nanocrystal samples at different temperatures. Reproduced with permission from Ref. [44]. Copyright 2011 American Chemical Society; **d1** TEM image and **d2** HRTEM images of non-confined ultrafine MgH_2_. **d3** TGA curves of bulk MgH_2_ and non-confined ultrafine MgH_2_. **d4** Hydrogenation profile with temperature of bulk MgH_2_ and non-confined ultrafine MgH_2_, and isothermal hydrogenation curves of non-confined ultrafine MgH_2_. **d5** Comparison of the energy barriers for the hydrogen absorption and desorption of bulk MgH_2_ and non-confined ultrafine MgH_2_. Reproduced with permission from Ref. [78]. Copyright 2021 Royal Society of Chemistry

Experimentally, preparation of Mg/MgH_2_ nanoparticles seems to be difficult, due to the high reactivity of Mg. In 2008, Aguey-Zinsou et al. [75] successfully prepared surfactant-stabilized Mg nanoparticles with an average diameter of 5 nm through electrochemical synthesis, which exhibited unique hydrogen storage properties (Fig. 4a). Almost all hydrogen could desorb from colloidal MgH_2_ at a low temperature of 85 °C. This was the first time that hydrogen desorption near room temperature was achieved by nanosized MgH_2_. Except for MgH_2_/Mg nanoparticles, Chen et al. innovatively reported the non-confined Mg nanoparticles with different morphologies (nanowires, nanoflakes, nanorods, and sea-urchin-like shapes) via a vapor-transport method, which displayed enhanced hydrogen-sorption kinetics (Fig. 4b) [76, 77]. However, unsatisfactorily, the minimum diameter of these nano-sized Mg was larger than 30 nm. Mg nanocrystals of controllable sizes smaller than 30 nm were successfully synthesized in gram quantities by Norberg et al. [44] through chemical reduction of magnesocene using a reducing solution of potassium (Fig. 4c). The prepared Mg nanocrystals with smaller diameters exhibited dramatically faster hydrogen sorption kinetics, attributed to the reduction of particle size and increase of the defect density.

**Fig. 5:**
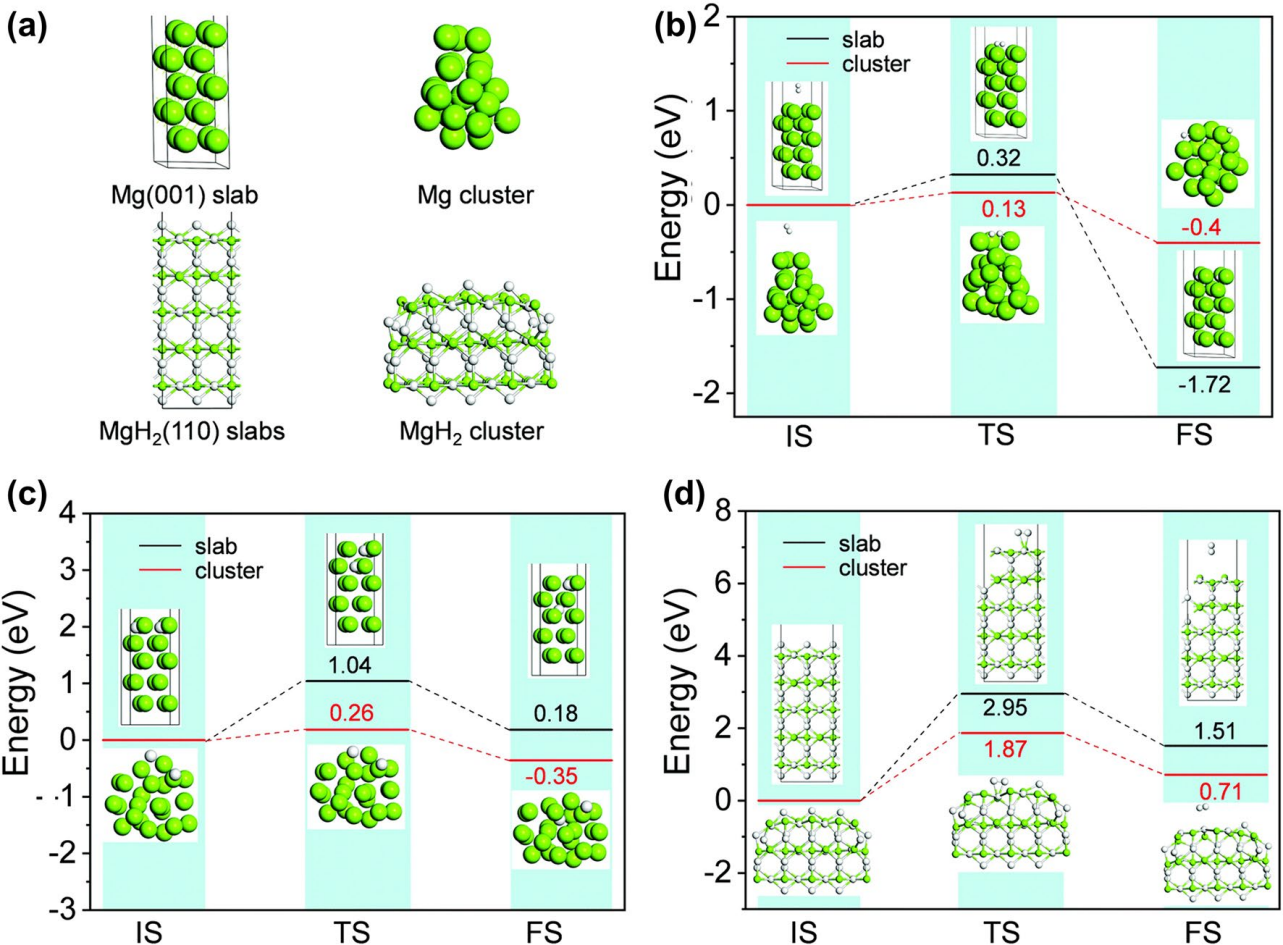
**a** Computational structure models for bulk and nanosized Mg and MgH_2_. **b** Hydrogen uptake by Mg (001) slab and Mg cluster. **c** Diffusion energy barrier of H atoms in the Mg (001) slab and Mg cluster. **d** Hydrogen release from MgH_2_ (110) slab and MgH_2_ cluster. Reproduced with permission from Ref. [78]. Copyright 2021 Royal Society of Chemistry. (IS: initial state, TS: transition state, FS: final state. Mg: green. H: white). (Colour figure online)

However, unfortunately, it is difficult to synthesize free-standing ultrafine MgH_2_ nanoparticles (< 10 nm) due to their high surface energy, strong reduction trend, and high water–oxygen sensitivity. The particle size of MgH_2_ synthesized by the methods mentioned above is generally large (> 30 nm) and the improvements in hydrogen storage performance are limited, unable to meet the requirements of practical applications. Recently, Zhang et al. [78] synthesized non-confined ultrafine MgH_2_ nanoparticles (4–5 nm) by the metathesis process of liquid–solid phase driven by ultrasound (Fig. 4d). The ultrasound was used to provide the driving force for the formation of MgH_2_, while combining the mechanical oscillation generated by ultrasound to inhibit the agglomeration of particles. A reversible hydrogen storage capacity of 6.7 wt% at 30 °C was achieved, bringing MgH_2_ a step closer to practical applications. Furthermore, the results of DFT calculations revealed that the reaction barrier for the decomposition of nano-sized MgH_2_ was remarkably lower than that of bulk MgH_2_ (Fig. 5), indicating that nanostructure engineering of MgH_2_ is thermodynamically and kinetically favorable to the enhanced performance.

### Preparation of Nanostructured Mg-Based Composites via Mechanical Milling

Mechanical milling plays a significant role in the field of energy storage and conversation, such as batteries, catalysis and hydrogen energy. Particularly, ball-milling has been proven to be a facile method to prepare Mg-based composites in hydrogen storage fields. It has been found that the MgH_2_ and catalysts with various morphologies and compositions can be mixed evenly to form nanostructured Mg-based composites through the simple ball-milling method. Impressively, plentiful defects and nanocrystalline are ingeniously generated during the process of high-energy ball milling. The introduction of defects and reduction of particle size could provide more active sites for de/re-hydrogenation and shorten hydrogen diffusion paths, thereby improving the hydrogen ab/de-sorption kinetics.

As early as the late 1990s, Huot et al. [79] first prepared MgH_2_ nanocrystals by using ball milling method. They found that the specific surface area of MgH_2_ increased from 1.2 to 9.9 m^2^ g^−1^ after ball milling, due to the reduction of particle size. However, the simple ball milling showed no obvious improvement in the performances of MgH_2_. Usually, de/re-hydrogenation of MgH_2_ is a catalytic reaction process depending on the catalytic activity of catalysts. Therefore, most of the research is focused on the introduction of catalysts into MgH_2_ to fabricate nanostructured Mg-based composites via the ball-milling method. The combination of the disordered MgH_2_ structure induced by mechanical milling with catalysts gives rise to synergetic effects and excellent ab/de-sorption properties.

Numerous catalysts can be introduced into MgH_2_ system, including transition metals [33, 34, 70, 80, 81, 82, 83, 84], oxides [85, 86, 87, 88], carbides [89, 90, 91, 92, 93], nitrides [85, 94, 95], and halides [96]. These materials share some similarities. First, the 3d-metal additives could obviously reduce the activation energy of hydrogen desorption. Second, the transition metal catalysts could chemisorb hydrogen and accelerate the diffusion of hydrogen to the Mg matrix. Third, the interfaces created by the introduction of the catalysts could act as active nucleation sites for the hydride phase, which would reduce the nucleation barrier for MgH_2_. Due to the above-mentioned similarities, the formation of high-performance nanostructured Mg-based hydrogen storage composites during ball-milling is expected.

Liang et al. [80] proposed MgH_2_–TM (TM = Ti, V, Mn, Fe, Ni) nanocomposite powders through intensive mechanical milling. They found that the MgH_2_–Ti and MgH_2_–V nanocomposites exhibited rapid desorption kinetics above 523 K and absorption kinetics at temperature as low as 302 K. Furthermore, it is worth mentioning that the mechanically milled MgH_2_–Ni nanocomposite exhibited better absorption kinetics than that of Mg–Ni alloy, due to the smaller particle size and better distribution of Ni-catalyst.

**Fig. 6:**
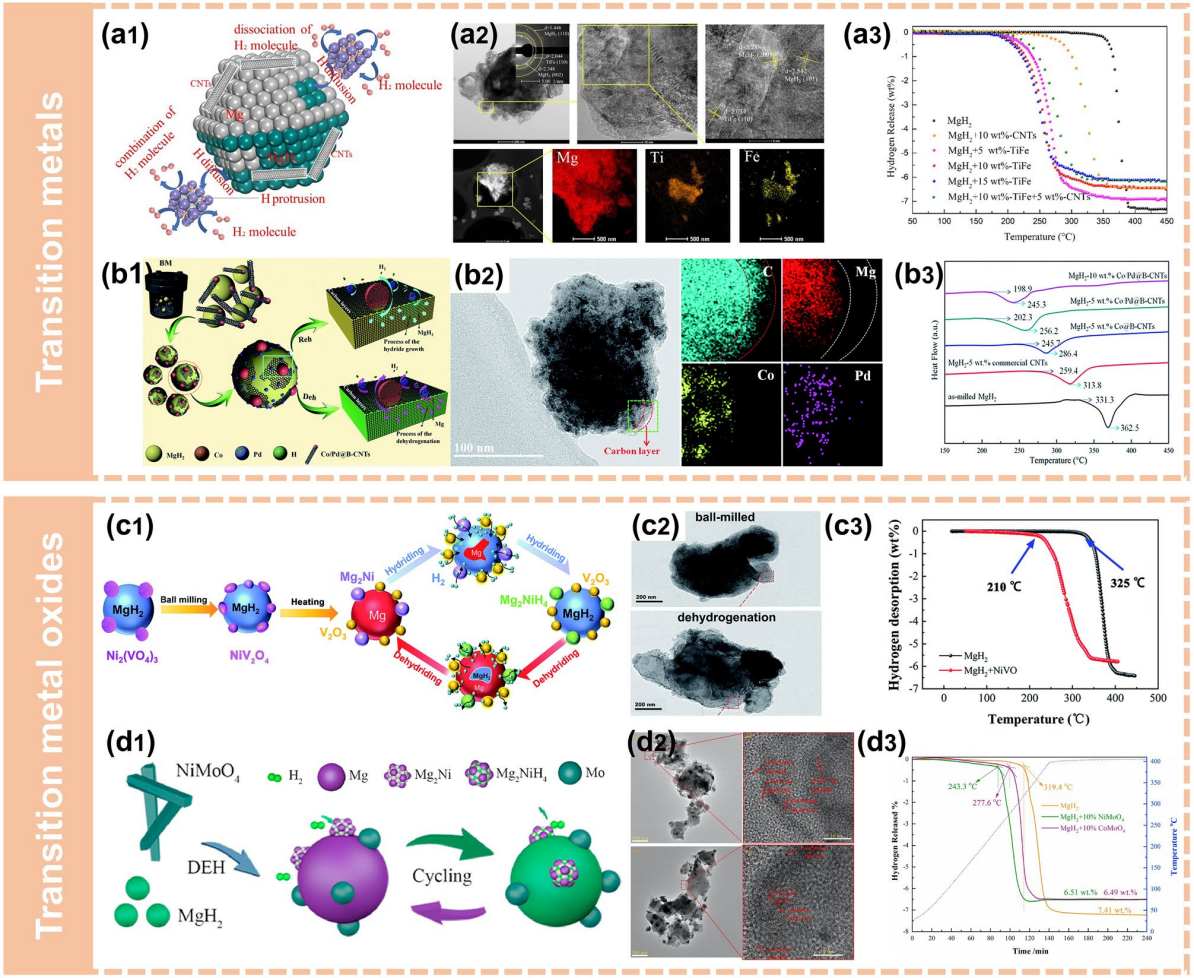
Synthesis of nanostructured Mg-based composites via mechanical milling. **a1** Schematic summary of the catalytic mechanism for the TiFe and CNTs catalyzed MgH_2_ particles. **a2** TEM images with corresponding SAED pattern and EDS mappings of the MgH_2_ + 10 wt%-TiFe composite. **a3** Non-isothermal desorption curves of different samples. Reproduced with permission from Ref. [84]. Copyright 2021 Elsevier; **b1** A schematic illustration of the "bidirectional catalysis" mechanism of Co/Pd@B-CNTs during the dehydrogenation and hydrogenation of MgH_2_. **b2** TEM images and EDS profiles of MgH_2_–10 wt% Co/Pd@B-CNTs after ten cycles. **b3** DSC curves of different samples. Reproduced with permission from Ref. [97]. Copyright 2019 Royal Society of Chemistry; **c1** Schematic diagram of hydrogen absorption and desorption process. **c2** TEM images. **c3** TPD curves. Reproduced with permission from Ref. [98]. Copyright 2021 Royal Society of Chemistry; **d1** A schematic illustration of the dehydrogenation and hydrogenation process of the MgH_2_–NiMoO_4_ system. **d2** TEM and HRTEM images of MgH_2_–10 wt% NiMoO4 after the 1st (top) and 10th (down) dehydrogenation. **d3** TPD curves. Reproduced with permission from Ref. [99]. Copyright 2021 Elsevier

Doping of nano-sized catalysts is crucial for enhancing the hydrogen storage performance of MgH_2_, but it is less satisfactory to develop a single catalyst to enhance both hydrogen desorption and absorption properties to a certain degree. Therefore, the combination of two or more metal catalysts could further achieve simultaneous modification of the hydrogen absorption and release kinetics of MgH_2_ [84, 97] (Fig. 6a, b). Liu et al. [97] introduced a bidirectional Co/Pd@B-CNTs into MgH_2_ through ball milling (Fig. 6b1, b2). As depicted in Fig. 6b3, the composites presented superior hydrogen desorption properties at relatively low temperatures. They proposed a special mechanism for "bidirectional catalyst" Co/Pd, in which Pd accelerated the diffusion of hydrogen during the absorption process and phase transformation between Mg_2_Co and Mg_2_CoH_5_ facilitated the release of hydrogen atoms.

Notably, the bimetallic oxides (e.g., Ni_3_(VO_4_)_2_ [98], TiNb_2_O_7_ [100], Ni/CoMoO_4_ [99]) have also been synthesized and applied to catalyze the re/de-hydrogenation of MgH_2_ via mechanical milling (Fig. 6c, d). The introduction of multi-element metallic catalysts into MgH_2_ by ball milling can not only make use of the synergistic effect from different metallic catalysts to enhance the hydrogen absorption and desorption performances of MgH_2_ at the same time, but also effectively promote the uniform distribution of multiphase catalysts. Huang et al. [99] investigated the influence of the bimetallic oxides NiMoO_4_ on the hydrogen storage properties of the MgH_2_ (Fig. 6d). They demonstrated that "Mg_2_Ni/Mg_2_NiH_4_" formed during hydrogen release/uptake process acted as "hydrogen pump" to boost the hydrogen storage performance of MgH_2_. In situ formed Mo^0^ not only weakened Mg–H bonding, but also further facilitated the mutual "Mg_2_Ni/Mg_2_NiH_4_" transformation (Fig. 6d1, d2). Ascribing to the collaborative action between Ni– and Mo– containing species, the hydrogen storage kinetics of MgH_2_ had been accelerated (Fig. 6d3).

To avoid growth and agglomeration of catalysts during re/de-hydrogenation at high temperatures, carbon-based materials are used to load catalysts to promote their dispersion. For example, amorphous carbon-embedded porous Nb_2_O_5_ [101], Ti_3_C_2_T_x_ supported Ni@C nanoflakes [89, 102, 103], carbon nanotube loaded multi-valence Co [34, 104], carbon-encapsulated ZrO_2_ [36] etc. were successfully synthesized and milled with MgH_2_ to improve hydrogen storage performances. It is worth emphasizing that the catalytic activity of catalysts loaded on carbon-based materials is much better than that of pure catalysts, due to the reduction of particle size, uniform distribution, and improvement of electrical conductivity.

**Fig. 7:**
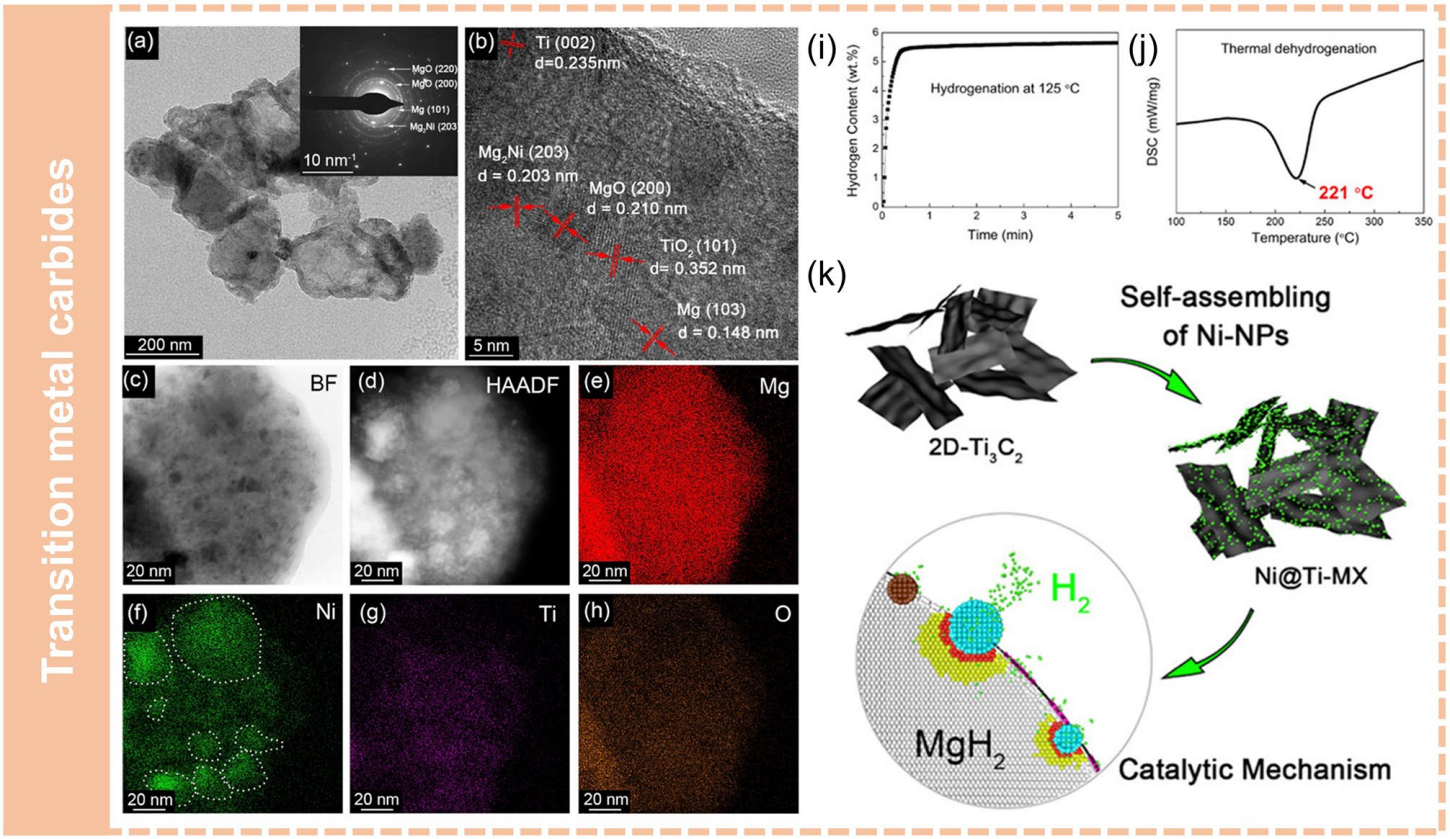
Synthesis of nanostructured Mg-based composites via mechanical milling. **a** BF-TEM image and the corresponding SAED pattern (inset). **b** HRTEM image, and **c–h** EDS mapping of the dehydrogenated MgH_2_ + Ni@Ti-MX composite. **i** Isothermal absorption curve of MgH_2_ + Ni@Ti-MX at 125 °C. **j** DSC profiles of MgH_2_ + Ni@Ti-MX at a heating rate of 3 °C min^−1^. **k** Schematic illustrations of the synthesis process of Ni@Ti-MX and the proposed mechanism for fast hydrogen ab/de-sorption of MgH_2_ + Ni@Ti-MX system. Reproduced with permission from Ref. [102]. Copyright 2020 American Chemical Society

Zhu et al. [102] synthesized a novel Ti_3_C_2_ MXene-based catalyst (Ni@Ti-MX) where ultra-dispersed Ni nanoparticles were anchored on the monolayer Ti_3_C_2_ flakes, and introduced into MgH_2_ through ball milling to fabricate MgH_2_ + Ni@Ti-MX nanocomposites (Fig. 7). Benefiting from the reduction of particle size during the ball milling process, the uniform dispersion of catalyst and the formation of multi-phase catalysts, the composites showed excellent performance. Hydrogen absorption at room temperature with a hydrogen storage capacity of 4 wt% was achieved. Particularly, the composites could absorb 5.4 wt% H_2_ in 25 s at 125 °C and the desorption peak temperature reduced to 221 °C (Fig. 7i, j).

It is no doubt that the fabrication of nanostructured Mg-based hydrogen storage composites by ball-milling is an efficient and simple method to improve the kinetics of MgH_2_. However, the nanoparticles generated by this method are typically large with an inhomogeneous size distribution, and extra impurities will be introduced. Moreover, the particles inevitably occur growth and self-aggregation during hydrogen cycling, resulting in hydrogen capacity decay. More importantly, the thermodynamics of Mg-based hydrogen storage composites prepared by ball-milling usually show no significant change, thus the desorption temperature of MgH_2_ cannot be lowered below that of the bulk (573 K). On the basis of this, a novel material synthesis method, namely dielectric barrier discharge plasma-assisted milling (P-milling) was developed by Ouyang et al. [105, 106], which realized the dual tuning of the thermodynamic and kinetic properties of MgH_2_. They reported that Mg(In)–MgF_2_ composite synthesized by P-milling technique exhibited a much higher decomposition equilibrium than pure MgH_2_ at the same temperature, which was caused by the rapid formation of a Mg-based solid solution [such as Mg(In)], confirming the thermodynamic destabilization. Meanwhile, the kinetics is also modified by the catalyzing effect of in situ formed MgF_2_ during P-milling process, thus the dual tuning of thermodynamic and kinetic properties is realized.

### Construction of Core–Shell Nanostructured Mg-Based Composites Through Chemical Reduction

Core–shell structures are of particular interest in the development of advanced composites as they can efficiently bring different components together at nanoscale. It has been widely employed in various fields including catalysis, energy, biology, and sensing. The advantage of this structure is greatly dependent on the distinctive synergistic effect between core and shell, thus obtaining a high-performance material.

Decoration of Mg nanoparticles with transition metal shells has become a widely used strategy to fabricate advanced nanostructured Mg-based hydrogen storage composites (Mg@TM) possessing potential advantages of encapsulation and catalysis effect originating from transition metal shells. The fabrication of Mg@TM core–shell structure could increase the contact area between the catalyst and hydrogen/matrix and provide more nucleation sites for MgH_2_/Mg, making full use of catalytic activity of the catalyst and improving hydrogen storage performance of MgH_2_. In addition, the strategy is simple, low-cost, and easy to scale up.

**Fig. 8:**
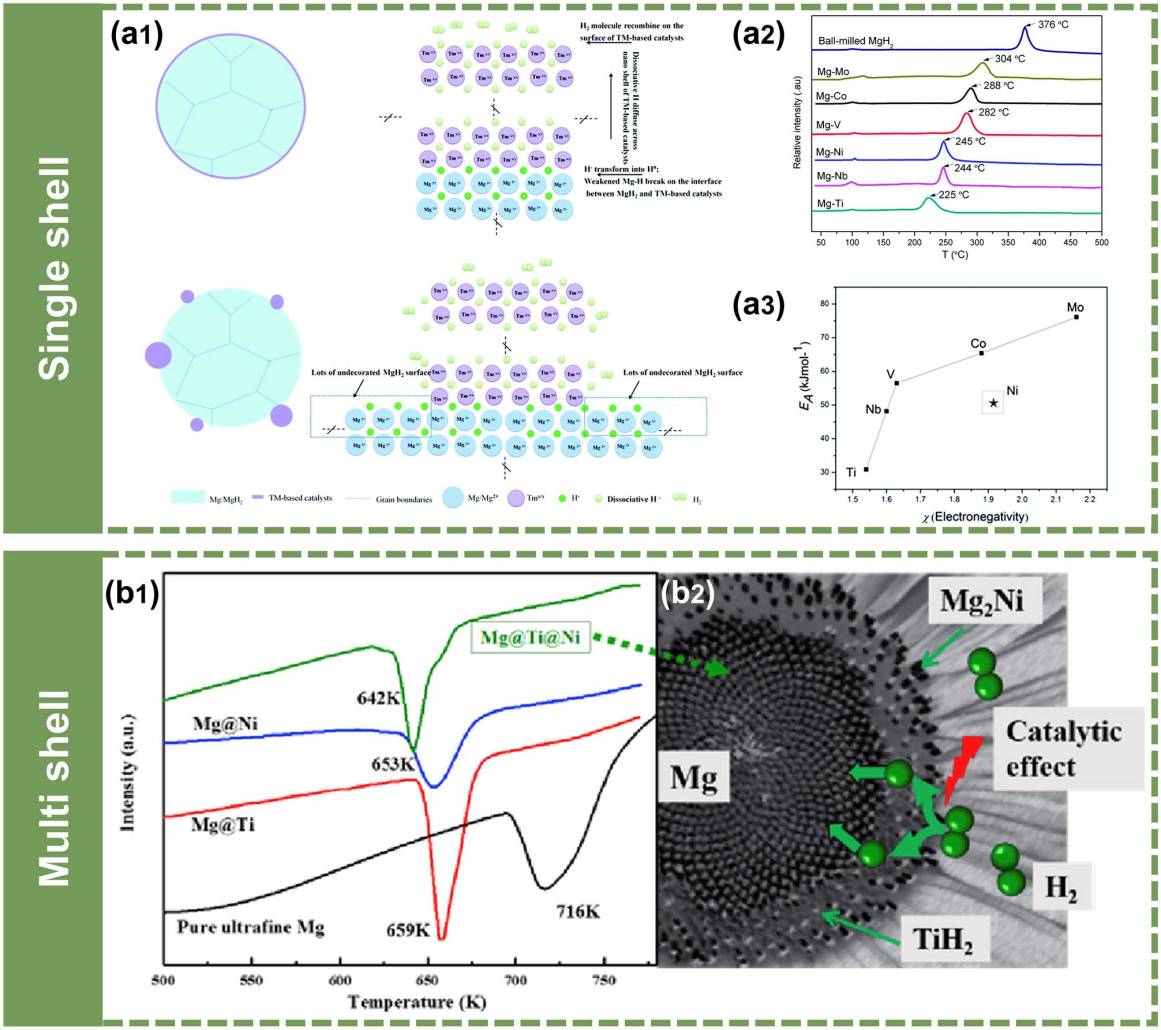
Synthesis of core–shell nanostructured Mg-based composites through chemical reduction. **a1** A schematic diagram of the Mg–TM and ball-milled Mg–TM-based catalysts. **a2** TPD-MS curves of Mg–TM samples with a heating rate of 4 K min^−1^. **a3** Plot of E_A_ verses χ of TM in Mg–TM systems. Reproduced with permission from Ref. [107]. Copyright 2014 Royal Society of Chemistry; **b1** DSC curves of different samples. **b2** A schematic diagram of Mg@Ti@Ni composite with core–shell structure. Reproduced with permission from Ref. [109]. Copyright 2017 Elsevier

As a successful example, Cui et al. [107, 108] reported the fabrication of a series of unique Mg–TM core–shell structures through a facile chemical reaction of the pre-milled Mg powder with the corresponding chlorides. They found that the composition of the shell can be modulated from Ti to Nb, V, Co, Mo, and Ni by simply changing the cation of the precursor (TiCl_3_, NbCl_5_, VCl_3_, CoCl_2_, MoCl_3_, NiCl_2_) (Fig. 8a). During the process of chemical reduction, a portion of Mg reacted with the TMCl_x_ to form a thin TM shell coating on the Mg core. This was completely different from the point contact of Mg/MgH_2_ with the TM-based catalyst by ball-milling (Fig. 8a1). The results showed that composites can desorb hydrogen even under 200 °C (Fig. 8a2). Particularly, the dehydrogenation performance was in the sequence of Mg–Ti, Mg–Nb, Mg–Ni, Mg–V, Mg–Co, and Mg–Mo, due to the decrease in electro-negativity from Ti to Mo (Fig. 8a3).

As another example, Lu et al. [110] first synthesized a core–shell nanostructured Mg@Pt composite, consisting Mg particles as the core with nano-sized noble metal Pt particles distributed homogeneously on Mg ultrafine particles. According to the in-situ TEM observations and DFT calculation, they creatively demonstrated a new mechanism that the H-stabilized Mg_3_Pt/Pt acted as the "hydrogen pump" for the dehydrogenation of MgH_2_ to improve the dehydriding kinetics of the hydrogenated Mg@Pt composite, attributed to the unique core–shell structure of Mg@Pt.

Lu et al. [109] extended the chemical reduction strategy for the fabrication of multi-shelled ternary Mg@Ti@Ni composites (Fig. 8b). The synthesis process of Mg@Ti@Ni core–shell structure included two-step electroless plating, namely, Cp_2_TiCl_2_ and NiCl_2_ *n*-butyl were added to THF solution of Mg powder in sequence. According to the chemical reactions, they successfully prepared a ternary Mg@Ti@Ni core–shell structure. Attributing to the co-effect of TiH_2_ acting as the activation site and Mg_2_Ni acting as the "hydrogen pump", the composites exhibited excellent thermodynamic performance and accelerated hydrogenation kinetics.

### Fabrication of Multi-dimensional Nano-sized Mg-Based Heterostructure Through Nanoconfinement Method

The nano-confinement method has been widely employed in the preparation of stabilized nanoparticles in the fields of energy storage and conversation. Recently, nano-confinement has been further proven to be an efficient process in constructing nanostructured Mg-based hydrogen storage composites.

Nano-confinement is the process in which a stable scaffold is acted as the substrate to support/confine nanoparticles/nanoclusters, inhibiting the growth and self-aggregation of the host [42, 43, 111]. The common features of these support materials are that they all possess high specific surface area and abundant porosity, providing sufficient nucleation sites for Mg/MgH_2_. As a result, the growth and self-aggregation of nano-sized particles are conspicuously avoided, keeping an excellent nano-sized effect (such as more exposed active sites, high specific surface area, and shortened diffusion pathway) and ensuring stable and excellent hydrogen storage performances. Melt impregnation and liquid impregnation are two commonly used methods to achieve nanoconfinement of MgH_2_/Mg.

The Mg precursor most widely used for the infiltration procedure is di-*n*-butyl-magnesium (MgBu_2_) or bis-cyclopentadienyl magnesium (MgCp_2_) [112], and then they were converted to MgH_2_/Mg under hydrogen/argon atmosphere at high temperatures based on the following reactions:

3-2 $${\text{MgBu}}_{2} + {\text{ H}}_{2} \to {\text{MgH}}_{2} + {\text{ C}}_{4} {\text{H}}_{10}$$

3-3 $${\text{MgCp}}_{2} \to {\text{Mg }} + {\text{ C}}_{5} {\text{H}}_{5}$$

According to different operating conditions, the hydrogenation method of MgBu_2_ can be divided into solid-state hydrogenation and liquid-state hydrogenation. The former refers that the hydrogenation process is performed after the solvent was completely evaporated. The procedure is described as follows: scaffolds are dispersed in MgBu_2_ solution in Ar filled glove box; when the solvent (heptane) is completely evaporated, MgBu_2_ is loaded onto the scaffolds; then the dried composites are transferred to the PCT reactor for further hydrogenation under the condition of 45 bar hydrogen pressure at 200 °C for 12 h. In terms of the liquid-state hydrogenation process, MgBu_2_ is converted to MgH_2_ through a solvothermal strategy under hydrogen atmosphere, and then solvent is completely evaporated to obtain MgH_2_@scaffolds nanocomposites.

As early as 2005, Liang et al. [113] first suggested that the hydrogen desorption enthalpy for MgH_2_ confined in carbon nanotube may be lowered and such confinement effect is independent of the tube length by theoretical calculations. Jongh et al. [114] successfully prepared Mg nano-crystallites by infiltration of nano-porous carbon with molten magnesium. Surprisingly, the Mg nanoparticles with a size smaller than 5 nm were first synthesized, which was never achieved before by unsupported nano-sized Mg/MgH_2_. This is the first experimental study about the influence of both nanosizing and support interaction on hydrogen sorption properties of MgH_2_.

**Fig. 9:**
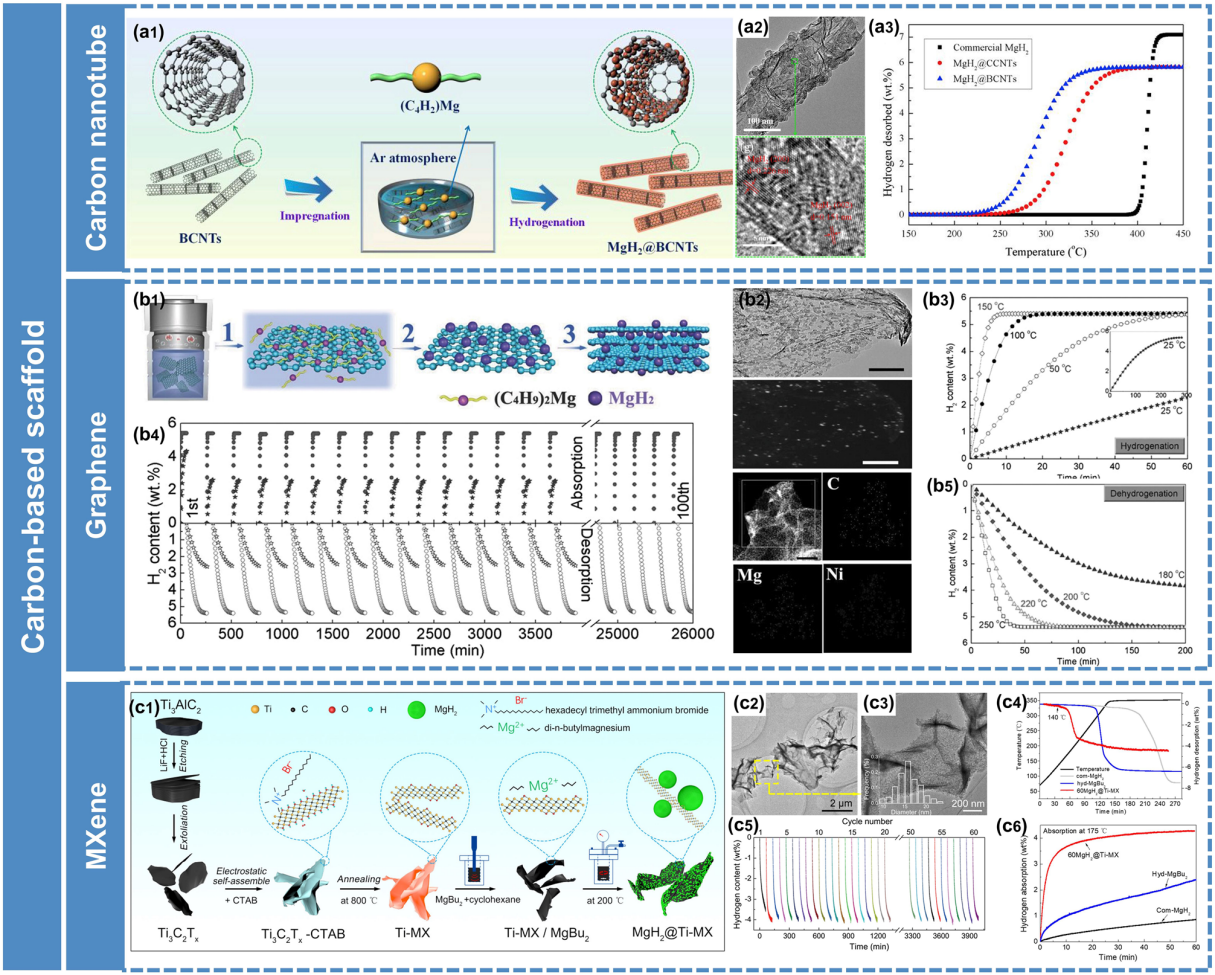
Synthesis of nanostructured Mg-based composites through the nano-confinement method. **a1** Schematic illustration of the self-assembly of MgH_2_ NPs on the BCNTs. **a2** Representative TEM and HRTEM images of MgH_2_@BCNTs. **a3** TPD curves of different samples. Reproduced with permission from Ref. [115]. Copyright 2019 Elsevier. **b1** Schematic illustration of the self-assembly of MgH_2_ NPs on GR. **b2** TEM, STEM images of Ni-MHGH-75, and corresponding elemental mapping images. **b3** Hydrogenation and **b5** dehydrogenation of Ni-MHGH-75 at various temperatures. **b4** Reversible H_2_ absorption and desorption of Ni-MHGH-75 (circles) and MHGH-75 (stars) at 200 °C. Reproduced with permission from Ref. [116]. Copyright 2015 John Wiley and Sons; **c1** Schematic illustration of the fabrication of MgH_2_@Ti-MX. **c2, c3** TEM images of the 60MgH_2_@Ti-MX. **c4** TPD curves of different samples. **c5** Cycling dehydrogenation curves of 60MgH_2_@Ti-MX at 200 °C. **c6** Isothermal hydrogenation curves of different samples. Reproduced with permission from Ref. [117]. Copyright 2021 American Chemical Society

To prevent excess MgBu_2_ from sticking on the surface of the scaffold material, a multistep impregnation approach was developed for the fabrication of nano-confined Mg/MgH_2_ [30, 115]. Zhao-Karger et al. [30] synthesized MgH_2_ particles smaller than 3 nm confined in a carbon scaffold by repeating the procedures of impregnation/drying with 1.5 mL of MgBu_2_ solution for each step along with the subsequent hydrogenation of MgBu_2_. Liu et al. [115] first used the novel 1D bamboo-shaped carbon nanotubes (BCNTs) as carriers for the self-assembly of MgH_2_ nanoparticles (15–20 nm) via repeating the procedures of impregnation/drying/hydrogenation three times (Fig. 9a). As a result, the extremely high loading capacity (76.8 mass fraction) was achieved and the obtained MgH_2_@BCNTs showed remarkably improved thermodynamics and kinetics for H_2_ adsorption and desorption.

It should be pointed out that, in a broad sense, if we define the shell structure composed of transition metal in core–shell nanostructured Mg@TM as an "encapsulation layer", construction of core–shell structure through chemical reduction can be regarded as a confinement approach as well. The only difference is that the encapsulation layer, which can also prevent Mg/MgH_2_ nanoparticles from growing up and agglomerating, is introduced later for the synthesis of core–shell structure.

Carbon-based materials have long been expected as promising supports for constructing nano-sized hydrogen storage materials, due to their excellent stability and high specific surface area. Xia et al. [116] reported that monodispersed MgH_2_ nanoparticles were assembled on the graphene decorated with Ni, exhibiting remarkable hydrogen storage properties (Fig. 9b). However, graphene is susceptible to the interlaminar stacking due to the high π–π bonding energy and the strong physical cross-linking. Moreover, due to the inert surface of pure carbon materials, premodification is needed before confining metal hydrides, which is complex and may introduce pollutants or even impair the mechanical properties of carbon-based materials.

With the preceding understanding of the scaffold of nanoconfinement, intuitively, the novel materials, such as MXenes, MOF, and transition metal compounds, which possess high specific surface area and intrinsic catalytic activity, could also act as superior scaffolds to support MgH_2_ nanoparticles. Hence, our group used Ti_3_C_2_, Ni–MOF, MOF derivatives, TiO_2_ nanosheets, etc. to fabricate the multi-dimensional Mg-based heterostructure to address the inert deficiency of pure carbon materials.

As promising 2D materials, MXenes have been widely investigated due to their special layered structure, excellent catalytic activity, high conductivity, and good flexibility [118, 119]. Numerous studies have focused on the introduction of MXenes as simple catalysts into MgH_2_ through mechanical milling to improve kinetic properties [120]. However, simple ball-milling leads to the serious destruction of two-dimensional structure of MXenes, and their unique structural advantages cannot be fully exploited. By making full use of the structural advantages of MXenes, Zhu et al. [117] ingeniously designed 3D Ti_3_C_2_T_x_ composed of folded Ti_3_C_2_T_x_ nanosheets, and then used it as a scaffold to disperse MgH_2_ nanoparticles to construct MgH_2_@Ti-MX nanocomposites through a solvothermal strategy (Fig. 9c). The obtained composites exhibited excellent hydrogen ab/de-sorption kinetics and low hydrogen desorption temperature (T_onset_ = 140 °C), remarkably superior to that of the MgH_2_ systems confined by pure carbon-based materials, attributed to the nano-size effect and the in situ formed catalytic TiH_2_.

In addition to MXene, metal–organic frameworks (MOFs) based materials, including pristine MOFs, MOF composites, and their derivatives have attracted great attention as potential hydrogen storage materials. Due to their high specific surface area and unique porous structure, most of the researches on MOFs in the field of hydrogen storage have focused on physical adsorption. In 2012, a hybrid hydrogen storage material composed of MOF (SNU-90) and Mg nanocrystals was first reported, which could store H_2_ by both physisorption and chemisorption [121].

The nano-confinement of MgH_2_ in the mesoporous structure of a Ni-based metal–organic-framework (denoted as MgH_2_@Ni–MOF) was also successfully conducted with MgBu_2_ as precursor via a combination of solvothermal method and wet impregnation method followed by hydrogenation [125]. The results showed that MgH_2_@Ni–MOF nanocomposites possessed rapid kinetic properties and low hydrogen ab/de-sorption enthalpy, which was attributed to the synergistic nano-size effect of nanoconfined Mg/MgH_2_ in Ni–MOF and catalytic effects from in situ formed Mg_2_Ni/Mg_2_NiH_4_. Notably, this is the first attempt that scaffold itself not only acts as an "aggregation blocker" to impede the growth and agglomeration of Mg/MgH_2_, but also has excellent catalytic activity on the hydrogen sorption of MgH_2_/Mg.

**Fig. 10:**
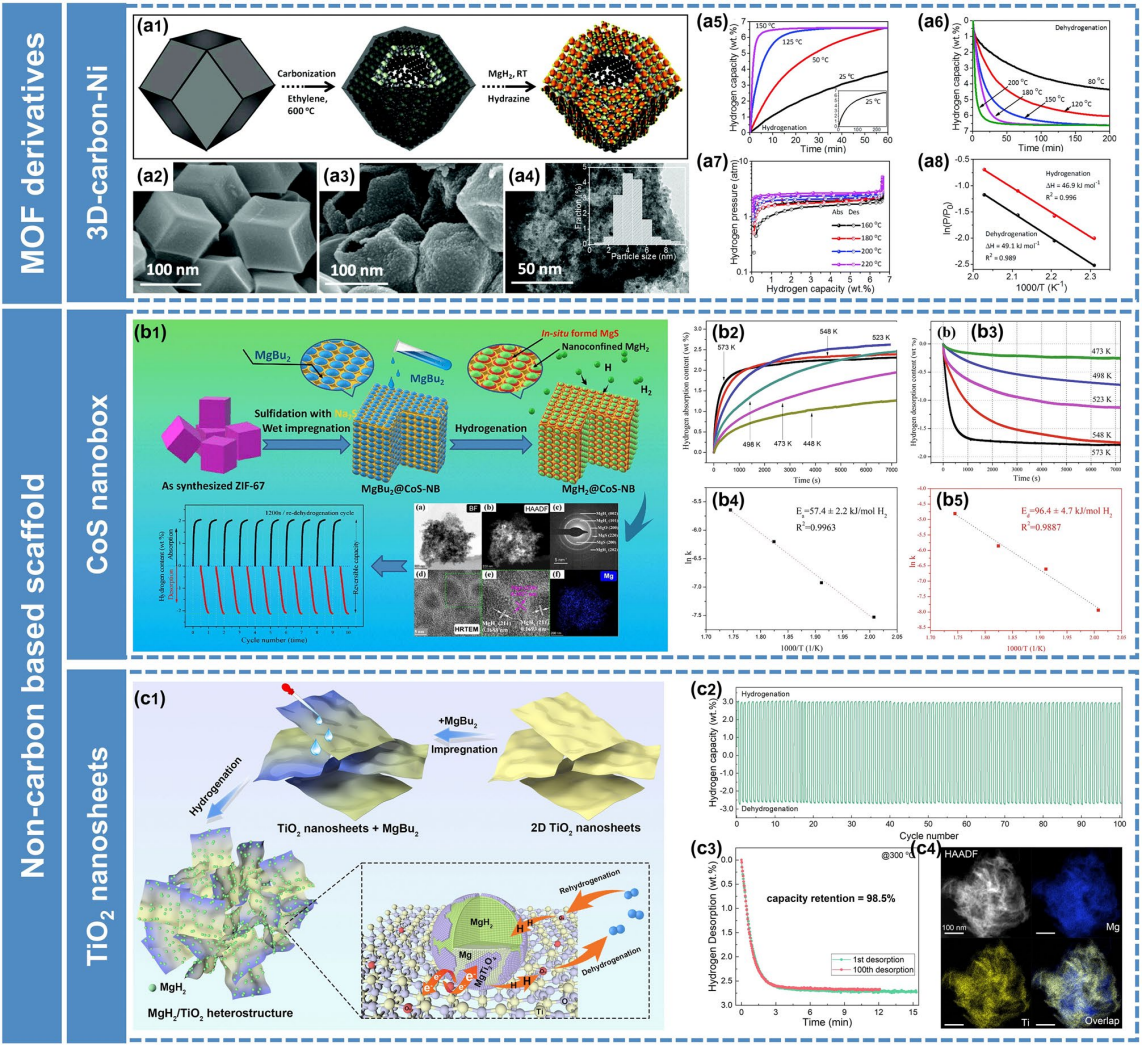
Synthesis of nanostructured Mg-based composites through nano-confinement method. **a1** Schematics displaying the self-assembled MgH_2_ on 3D metal interacted carbon. **a2** SEM image of the prepared metal-interacted 3-D carbon. **a3** SEM and **a4** TEM images of the MHCH-5. **a5** Hydrogen absorption and **a6** desorption of the MHCH-5 at different temperatures. **a7** Pressure–composition–temperature (PCT) plots of the MHCH-5 at different temperatures. **a8** The van't Hoff plots of the MHCH-5 derived from de-/hydrogenation. Reproduced with permission from Ref. [122]. Copyright 2017 Royal Society of Chemistry; **b1** Schematic illustrations of the nanoconfinement process for the synthesis of MgH_2_@CoS-NBs composite, TEM images of MgH_2_@CoS-NBs, and cycling behavior of the MgH_2_@CoS-NBs composite. **b2** Isothermal hydriding and **b3** dehydriding profiles, and the corresponding *lnk* verses 1000/*T* plots for determining the activation energy of **b4** hydrogenation and **b5** dehydrogenation. Reproduced with permission from Ref. [123]. Copyright 2021 Elsevier; **c1** synthesis process of MgH_2_/TiO_2_ composites and schematic diagram showing the hydrogenation and dehydrogenation mechanisms of MgH_2_/TiO_2_ heterostructure. **c2** Cycling profiles of 60MgH_2_/TiO_2_ at 300 °C. **c3** Hydrogen desorption curves of 60MgH_2_/TiO_2_ at different cycles. **c4** HAADF images of 60MgH_2_/TiO_2_ after 100 cycles and corresponding EDS elemental mapping results. Reproduced with permission from Ref. [124]. Copyright 2022 Springer Nature

However, micropores of pristine MOFs always hinder the impregnation of MgBu_2_ and the diffusion rate of H_2_ through the pores, resulting in a low loading capacity of MgH_2_ and limited improvement in performance. So far, huge attention and efforts have been paid to MOF derivatives [126]. Shinde et al. [122] presented a simple and scalable strategy to fabricate air-stable MgH_2_ embedded in MOF-derived 3D activated carbon with periodic synchronization of transition metals (MHCH) (Fig. 10a). The resulting MHCH-5 (TM = Ni) exhibited excellent hydrogen storage properties, with a high storage capacity of 6.63 wt% H_2_, rapid kinetics loading in < 5 min at 180 °C, superior reversibility, and excellent long-term cycling stability over ∼ 435 h. They demonstrated that the synergetic effects of nanoconfinement, uniform distribution of MgH_2_ NPs, exceptional structural and chemical stabilities, and high thermal conductivity contributed to the enhancement of hydrogen storage performance of MgH_2_. In order to increase the load capacity of MgH_2_ on the scaffold, Ma et al. [123] designed a novel CoS-nanoparticle-assembled nano boxes (denoted as CoS-NBs) scaffold derived from ZIF-67 with suitable mesoporous structure through a template-consumption method. And then MgH_2_ was confined within the porous structure of CoS-NBs (referred to as MgH_2_@CoS-NBs) by a wet impregnation method followed by hydrogenation at elevated temperatures (Fig. 10b). Benefiting from the appropriate mesoporous structure and excellent catalytic activity of CoS, the load capacity of MgH_2_ was up to 42.5 wt% and the hydriding/dehydriding enthalpies reduced to − 65.6/68.1 kJ mol^−1^ H_2_. Based on the structural designing strategies of MOFs, Zou et al. [127] further constructed a MOF-derived 1D *N*-doped hierarchically porous carbon nanofiber and used it as the scaffold for self-assembly of MgH_2_/Ni nanoparticles.

Besides the scaffolds mentioned above, we have recently discovered that 2D transition metal oxides (TiO_2_) nanosheets are also promising frameworks to host MgH_2_ nanoparticles (Fig. 10c) [124]. The results demonstrated the significant contribution of the nanoconfinement effect and Mg–Ti oxides to re/de-hydrogenation performances. It was worth emphasizing that abundant oxygen vacancies were introduced into TiO_2_ nanosheets during the impregnation process due to the strong reducibility of MgBu_2_, which had a positive influence on the improvement of MgH_2_.

Based on the above review, we concluded that significant breakthroughs in nanostructured Mg-based hydrogen storage materials were made in recent years, and their application for stationary/on-board hydrogen storage would be within sight.

## Promising Applications of Nanostructured Mg-Based Hydrogens Storage Materials

**Fig. 11:**
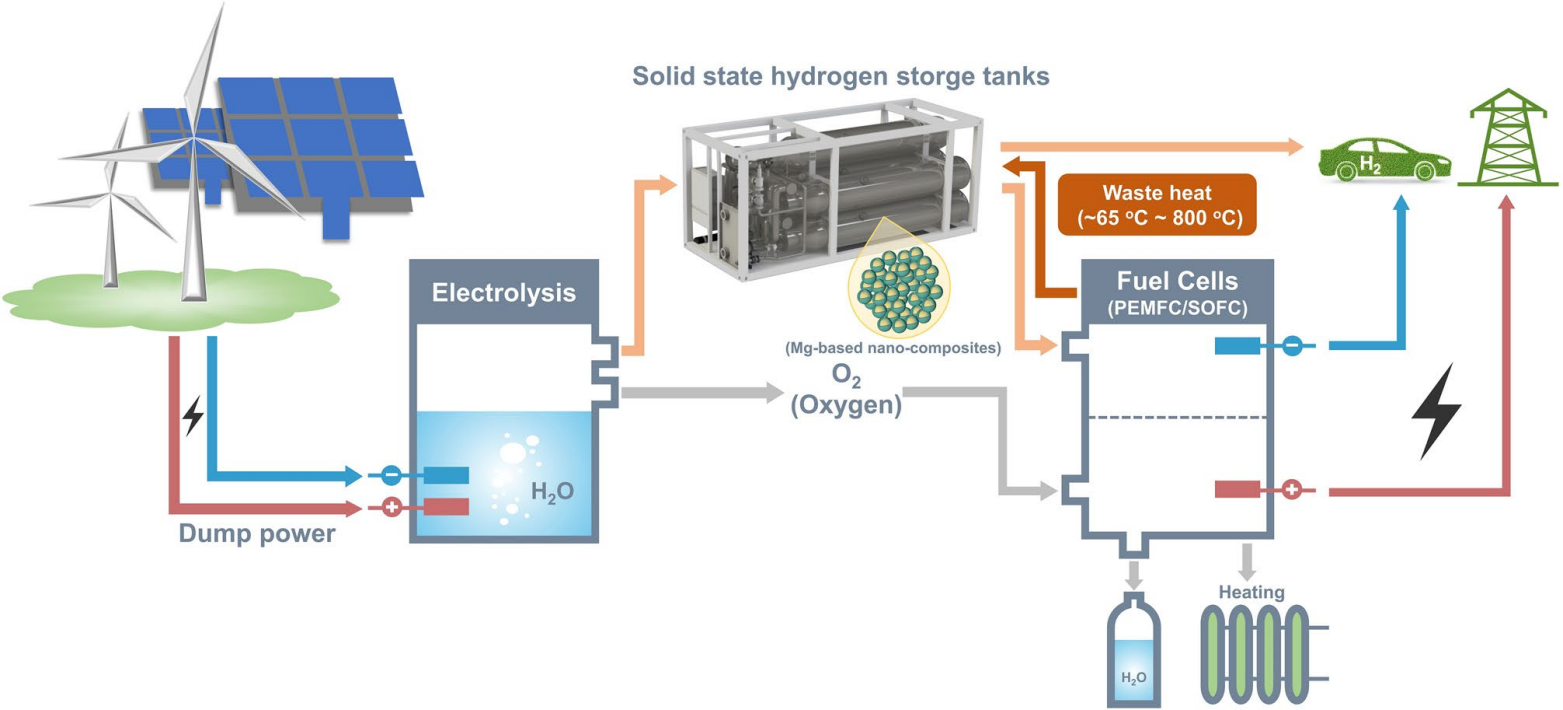
A vision of the future energy infrastructure (electrolysis system; safety design for the hydrogen storage and transportation device; construction of hydrogen system-linked hydrogen vehicles/fuel cell equipment charging system)

We have a bright vision of the new energy structure of "renewable energy for hydrogen production—hydrogen storage—transportation integration" in the future (Fig. 11). Hydrogen can be produced by electrolysis of water using electricity generated from clean energy sources, and the hydrogen is stored in a Mg-based hydrogen storage tanks. This system could supply high-purity hydrogen gas to a fuel cell for electricity generation or other industry usage at the same time. In turn, waste heat generated by PEMFC/SOFC can be used to provide energy for continued hydrogen release from the hydrogen storage tanks.

Compared with LaNi_5_-based materials and Ti–V(Cr)-based alloys with body-centered cubic structures, MgH_2_ has the promising potential to satisfy technical requirements for both gravimetric and volumetric capacities. Moreover, considering the cost and energy density of MgH_2_, it is very promising for small-scale portable applications to supply hydrogen through the hydrolysis reaction in water [128, 129]. Notably, nanostructured MgH_2_/Mg will improve the performances of the hydrolysis reaction, making it more efficient and convenient for small-scale portable applications.

Furthermore, MgH_2_ has the advantages of large heat storage density (3060 kJ kg^−1^), excellent reversibility, and low cost, which is available for large-scale heat storage to store excess heat from solar power plants or industries [130]. This is one of the hot research topics in chemical heat storage. In the daytime, excess solar energy can be used to promote the decomposition of MgH_2_, and the hydrogen can be stored in the hydrogen tank or low-temperature hydrogen-storage materials. At night, a heat energy density of 0.9 kWh kg_Mg_^−1^ will be released through the hydrogen absorption in Mg. It is noteworthy that additional catalysts need to be introduced into the MgH_2_/Mg system to improve its thermal storage efficiency; the cost of this part is difficult to estimate. If the synthesis process for the low-cost and large-scale nanosized Mg-based hydrogen storage materials can be developed, significant cost savings for thermal storage systems will be achieved through the use of nanostructured Mg-based hydrogen storage materials.

Impressively, nanostructured Mg/MgH_2_ can also be used as reactant of integrated Li(Na)BH_4_ hydrogen production/storage technology to maximize the regeneration efficiency of Li(Na)BH_4_ [131, 132, 133], which is the new topic for hydrogen-energy chain and hydrogen economics. What's more, nanostructured Mg-based hydride has also been successfully used as an advanced battery anode material for lithium-ion storage [134, 135, 136]. Indubitably, nanostructured Mg-based hydrogen storage materials will play a critical role in the future energy structure.

## Conclusion and Outlook

Herein, we have highlighted the most important synthetic methodologies and hydrogen storage applications of nano-structured Mg-based composites. Significant advances have been made in the fabrication of nanostructured Mg-based hydrogen storage materials in the last decades, accompanied by several breakthroughs in hydrogen storage performances toward industrial applications. Especially, near room temperature reversible hydrogen ab/de-sorption (30 °C) with considerable high capacity (> 6.7 wt%) has been achieved. Additionally, dual regulation of nanoconfinement and nano-catalysis has been realized. Moreover, the low ab/de-sorption temperature achieved by modified MgH_2_ has fallen into the range of proton exchange membrane (PEM) fuel cells, which is a big step forward for the application of nanostructured Mg-based hydrogen storage composites in the field of transportation. What's more, nanostructured Mg-based hydride has been successfully used as an advanced battery anode material for lithium-ion storage.

It is well known that all modifications of MgH_2_ have the same goals, i.e., lower working temperature, higher hydrogen storage capacity, and better cycling stability with lower cost and easier production process. With our current knowledge, it is worth emphasizing that the improvement of the properties of MgH_2_/Mg towards theoretical capacity of 7.6 mass% (110 g L^−1^) below 100 °C and long-term cyclic stability depends upon the choice of appropriate synthetic approaches to enable the full control over both kinetics and thermodynamics. For future research in the synthesis and application of nanostructured Mg-based hydrogen storage composites, we believe that it should concentrate on the following aspects:

- Most of the conventional methodologies suffer from one or more of the following deficiencies: difficulty in precise control and lack of uniformity and universality. Typically, the thermodynamics will be modified first and then some catalysts will be added. Such an approach generally results in capacity loss and sometimes additional degradation of the hydrogen properties due to the lack of "compatibility" between the catalyst, the hydride, and the thermodynamic enhancer. Thus, new synthetic strategies need to be developed to achieve precise position control of alloying elements and catalysts in Mg/MgH_2_ lattice at the atomic level to maximize their effects while retaining the hydrogen storage capacity of hydride.
- Nanoconfinement strategy has the disadvantage of troublesome synthesis procedure, unfeasible for mass production, and high cost. In this regard, the development of facile, versatile, low-cost, and scalable methods for the production of high-quality nanostructured Mg-based hydrogen storage materials and precisely controlled structural parameters is of primary importance. And the most notable point is that the commonly used precursor of MgBu_2_ in the synthesis of nanoconfined MgH_2_ nanoparticles, generally has disadvantages such as complex synthesis process, being expensive, and being prone to oxidation. Hence, in order to simplify the synthesis process of nanoconfined MgH_2_/Mg with "clean" and hydrogen active surfaces, new Mg precursors with high safety, low cost, good solubility in common solvents, low decomposition temperatures, and/or low reduction potential should be synthesized and the design of effective organic stabilizer should be pursued.
- To date, most studies about the scaffold of nanoconfinement strategy have focused on stable carbon materials (such as carbon nanotube and graphene), while complex oxide and nitride frameworks have rarely been reported. Given their unique catalytic activity, the fabrication of oxide and nitride frameworks with high specific surface areas is of great importance. Moreover, a novel scaffold with nano-confinement capability for hydride, high catalytic activity, and physical hydrogen storage capacity is highly desired, so that unique nanostructured Mg-based hydrogen storage composites with high capacity and superior performances can be synthesized combining both physical and chemical hydrogen storage.
- Lots of nanostructured Mg-based hydrogen storage materials are prepared relying on the experiences of researchers. The rational design of Mg-based hydrogen storage materials for specific applications based on material genome engineering technology remains a great challenge and should be strengthened in the future.
- The catalytic mechanism of some catalysts for Mg-based hydrogen storage materials remains elusive. It would be very helpful to deepen the comprehension and understanding of the catalytic mechanism, especially from the atomic level, which would spur novel and powerful methodologies for the construction of high-performance Mg-based hydrogen storage materials.
- The new energy storage infrastructure of "renewable energy for hydrogen production—hydrogen storage—transportation integration" should be taken into account in the future. Moreover, effective thermal management is also critical to the application of nanostructured Mg-based hydrogen storage materials in the field of on-board hydrogen storage.

The breakthroughs in the construction of nanostructured Mg-based hydrogen storage composites have provided opportunities to tune their hydrogen storage properties. Although MgH_2_ has been extensively studied as one of the most promising solid-state hydrogen storage materials, its application in other energy fields has attracted little attention. Considering the low cost and unique phase transformation behavior, we expect to see a surge in the application of nanostructured Mg-based hydrogen storage materials in various energy fields, such as energy storage of renewable energy.

Lastly, it should be emphasized that the kinetic and thermodynamic properties of nanostructured Mg-based hydrogen storage composites are still far from the requirements for the on-board applications. There is still a long way to go for the commercial-scale production and practical application of such intriguing materials. Although we have summarized some recent advances on the synthesis of nanostructured Mg-based hydrogen storage composites, we would rather regard this review as an opening remark than a concluding remark. We are confident that other versatile and powerful synthetic methodologies for nanostructured Mg-based composites will be developed in the future. We also believe that nanostructured Mg-based hydrogen storage materials will have a place in the fields of energy storage and conversion.
